# Fecal microbiota transplants (FMT) of three distinct human communities to germ-free mice exacerbated inflammation and decreased lung function in their offspring

**DOI:** 10.1128/mbio.03764-24

**Published:** 2025-04-10

**Authors:** Ivon A. Moya Uribe, Hinako Terauchi, Julia A. Bell, Alexander Zanetti, Sanket Jantre, Marianne Huebner, S. Hasan Arshad, Susan L. Ewart, Linda S. Mansfield

**Affiliations:** 1 Comparative Enteric Diseases Laboratory, Michigan State University, East Lansing, Michigan, USA; 2 College of Veterinary Medicine, Michigan State University, East Lansing, Michigan, USA; 3 Michigan State University, East Lansing, Michigan, USA; 4 Comparative Medicine Integrative Biology Program, College of Veterinary Medicine, Michigan State University, East Lansing, Michigan, USA; 5 Department of Microbiology and Molecular Genetics, Michigan State University, East Lansing, Michigan, USA; 6 Department of Large Animal Clinical Sciences, Michigan State University, East Lansing, Michigan, USA; 7 Department of Statistics and Probability, Michigan State University, East Lansing, Michigan, USA; 8 Clinical and Experimental Sciences, Faculty of Medicine, University of Southampton, Southampton, United Kingdom; 9 Human Development and Health, Faculty of Medicine, University of Southampton, Southampton, United Kingdom; 10 The David Hide Asthma and Allergy Research Centre, Isle of Wight, United Kingdom; 11 NIHR Biomedical Research Centre, University Hospital Southampton NHS Foundation Trust, Southampton, United Kingdom; University of Texas Health Science Center, School of Public Health, Houston, Texas, USA

**Keywords:** fecal microbiota transplant (FMT), allergy, asthma, airway hyperresponsiveness (AHR), eczema, lung function, murine model

## Abstract

Despite explosive rise in allergies, little is known about early life gut microbiota effects on postnatal respiratory function. We hypothesized that *Enterobacteriaceae*-dominant gut microbiota from eczemic infants increases Type 2 inflammation and decreases lung function in transplanted mice, while *Bacteroidaceae*-dominant gut microbiota from non-eczemic infants is protective. Fecal microbiota transplants (FMT) from eczemic infants “Infant A” and non-eczemic infants “Infant B” were successfully transplanted into germ-free C57BL/6 mice, passing to offspring unchanged. Infant A and B, Adult C-human-derived (positive control), and Mouse (negative control) microbiotas all in C57BL/6 mice were tested for effects on airway function in nonallergic (phosphate-buffered saline [PBS]) and allergic (house dust mite [HDM]) conditions. Baseline lung mechanics in mice with human microbiotas (^HU^microbiota) were significantly impaired compared to Mouse microbiota controls (^MO^microbiota) with or without HDM; respiratory system resistance (Rrs) was increased (*P* < 0.05–*P* < 0.01), and respiratory system compliance (Crs) was decreased (*P* < 0.05–*P* < 0.01). ^HU^Microbiota mice showed a statistically significant impairment compared to ^MO^microbiota mice in lung parameters Rrs, Ers, Rn, and G at baseline, and at multiple methacholine (MCh) doses with baseline removed. Impairment manifested as increased small airway resistance and tissue resistance. HDM significantly elevated IL-4, eosinophils, lung inflammation, and mucus cell metaplasia, and decreased macrophages and lung function (*P* < 0.05) in mice of all microbiotas, yet each ^HU^microbiota produced distinct features. Infant B and Adult C mice had elevated basal levels of total IgE compared to ^MO^microbiota and Infant A mice (*P* < 0.05). In ^HU^microbiota mice given HDM, only Adult C had elevated IL-5 and IL-13 (*P* < 0.05), only Adult C and Infant B mice had elevated neutrophils (*P* < 0.05), and only Infant A had elevated lymphocytes (*P* < 0.01).

**IMPORTANCE:**

Fecal microbiota transplants (FMT) of three distinct human communities to germ-free mice exacerbated inflammation and decreased lung function in their offspring. Taxa formerly described to induce an allergic response (agonists) and pro-inflammatory taxa were abundant in ^HU^microbiotas compared to ^MO^microbiota controls, while taxa formerly described to reduce allergic responses (antagonists) and anti-inflammatory taxa were numerous in ^MO^microbiotas and low in ^HU^microbiotas. Thus, we largely rejected our hypotheses because data supported multiple pro-inflammatory allergy agonists functioning in a community-wide fashion to impair lung function in the absence of antagonistic anti-inflammatory taxa. Structure of ^HU^microbiotas played a key role in determining varied allergic responses and resulting lung impairment, yet, strikingly, all mice with ^HU^microbiotas had impaired lung function even in the absence of allergens. Using a comparative approach, we showed that composition of gut microbiota can alter innate/immune regulation in the gut-lung axis to increase baseline lung function responses and the risk of allergic sensitization.

## INTRODUCTION

Asthma is a chronic airway disease characterized by inflammation and airway hyperresponsiveness (AHR) with a US prevalence of 7.9% (1). Asthma is the most common chronic condition in children worldwide and may be preceded by eczema, wheeze, or atopy (2). Multiple factors are associated with its development, including genetics, mode of birth, childhood setting, early animal exposures, antibiotic use, and composition of gut or lung microbiota (3–15). AHR, a cardinal feature of asthma, occurs when airways constrict excessively in response to a direct stimulus, e.g., histamine or methacholine (MCh) acting directly on receptors of airway smooth muscle (ASM) or to indirect stimuli (allergen) (16). AHR can be present in both allergic and nonallergic asthmatic patients (17, 18) and degree of AHR is correlated with disease severity, often precedes asthma in children, and is a main therapeutic target for management of symptoms (19). Yet mechanisms underlying AHR are heterogeneous including genetic factors, ASM alterations, airway extracellular matrix component remodeling, and airway inflammation (19, 20). AHR is characterized by functional changes in airway resistance and lung elastic properties; these changes are commonly assessed by measuring lung function (19).

Specific microbiota or microbes have been linked to pathogenesis of allergies, including asthma, eczema, and food allergies (21). Development of respiratory microbiota depends heavily on exposures during the first few hours of life onward, which are dependent upon delivery mode, early environment, and infections (22–24). Tracheal aspirates soon after birth showed dominant *Firmicutes* and *Proteobacteria* with the presence of *Actinobacteria* and *Bacteroidetes* (25), while Bisgaard et al. showed that in 1-month-old infants, bacterial colonization of the airway hypopharyngeal region with *Streptococcus pneumoniae*, *Haemophilus influenzae*, or *Moraxella catarrhalis* was associated with later development of asthma (26). In the lower respiratory tract, *Proteobacteria*, e.g., *Haemophilus*, *Moraxella*, and *Neisseria*, were over-represented in asthma patients compared to non-asthmatic volunteers (27).

Gut-lung axis studies showed effects of gut microbiota on allergic airway disease. Reduced diversity of fecal bacterial microbiota in 1- or 12-month-old infants was associated with increased risk of allergic sensitization, allergic rhinitis, and peripheral blood eosinophilia at 5 years (28). Penders et al. showed increased *Clostridium* cluster I prevalence at ages 5 and 13 weeks was positively associated with development of atopic dermatitis (29). Likewise, increased asthma risk at 5 years was significantly associated with increased abundance of *Veillonella* and decreased abundance of *Roseburia*, *Alistipes*, and *Flavonifractor* in fecal microbiota at 1 year of age (30). Protective taxa deficits have also been implicated in asthma pathogenesis. Lynch et al. showed that abundant *Firmicutes* and *Bacteroidetes* bacteria reduced asthma risk in the inner-city high asthma prevalence Urban Environment and Childhood Asthma cohort (31). Arrieta et al. showed that decreased abundance of *Lachnospira*, *Veillonella*, *Faecalibacterium*, *Rothia* and fecal acetate and dysregulation of enterohepatic metabolites occurred in children developing asthma (22). Depner et al. showed that protective effects of farm exposures in reducing asthma prevalence were correlated with bacterial taxa predicted to produce butyrate (*Roseburia, Coprococcus*) and increased abundance of genes encoding butyryl–CoA:acetate–CoA-transferase (32). These studies showed microbiota composition influences allergic susceptibility.

Mouse models also support early life microbiota effects on allergic sensitization (33–35). Upregulation of allergic airway inflammation occurred in germ-free mice that was reversed by early commensal gut microbiota colonization 4 weeks before sensitization to ovalbumin (33). Germ-free or low-diversity gut microbiota mice developed high serum IgE and mast-cell-surface-bound IgE, leading to ovalbumin-induced systemic anaphylaxis that was prevented by early mouse specific-pathogen-free (SPF) microbiota (34). In limited-flora gnotobiotic mice, gut *Clostridia* regulated innate lymphoid cell function and intestinal epithelial permeability by an innate lymphoid cell-3 (ILC3) and IL-22-dependent mechanism to protect against allergen sensitization (35). Moreover, mice fed house dust from homes with dogs exhibited significantly reduced bronchial responsiveness and lung inflammation after both allergic challenge and inoculation with respiratory syncytial virus (36). Yet, knowledge gaps exist regarding specific taxa and mechanisms by which gut microbiota in early childhood influences development of asthma and allergic diseases (37).

We sought to provide proof-of-concept for transplanted ^HU^microbiota models for studying mechanisms underlying microbial effects on allergic asthma in early life. We showed association of *Veillonella-Escherichia-Shigella* in low-diversity infant gut microbiota with development of persistent eczema in early childhood (12), while high-diversity microbial communities with high *Bacteroides* abundance were associated with protection against eczema. As eczema often precedes asthma, this suggested a link between microbiota and asthma (38). We hypothesized that offspring of mice transplanted with *Veillonella-Escherichia-Shigella* microbiotas from infants with persistent eczema would develop allergic inflammation with a lower threshold to MCh-induced AHR when sensitized to house dust mite (HDM) and, conversely, mice with high levels of *Bacteroides* spp. would be protected. We conducted fecal microbiota transplants (FMT) from infants with persistent eczema and infants with no allergic manifestations into germ-free C57BL/6 mice. As a positive control for AHR after HDM, we used offspring from germ-free mice transplanted with microbiota from healthy young adults (Adult C) that showed a Type 2 immune bias to *Campylobacter jejuni* challenge (39, 40). Thus, using three ^HU^microbiota-transplanted mice and Mouse microbiota (^MO^microbiota) negative controls, all of the same C57BL/6 genetic background, we conducted a study to test effects of early microbiota composition on the development of AHR with and without HDM exposure.

## RESULTS

### Infant fecal FMT

**Fig 1:**
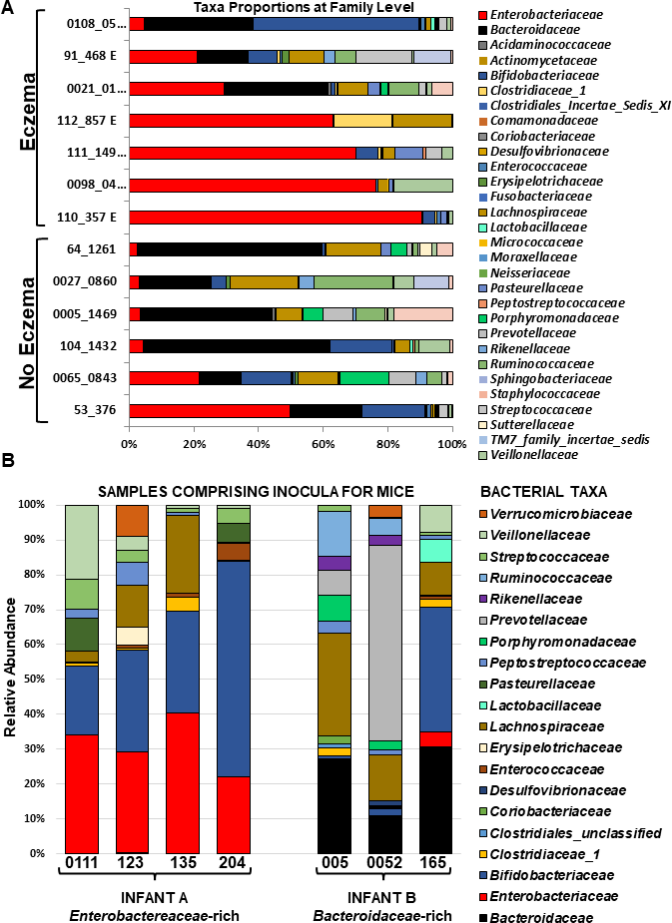
Relative abundance of fecal bacterial families and other taxa in Isle of Wight 3-month-old infants. (**A**) Proportions in eczemic and non-eczemic infant samples. (**B**) Infant samples mixed and used to inoculate two groups of germ-free mice.

Fecal samples from 60 3-month-old infants were analyzed in an ongoing multigenerational prospective longitudinal study of newborns (Isle of Wight [IOW] third generation birth cohort) using 16S rRNA gene sequencing to examine the association of bacterial taxa profiles with risk of eczema (12). Three-month-old infants harboring a fecal microbiota with increased abundance of *Escherichia coli-Shigella* and *Bifidobacterium* had higher risk for eczema at 1–3 years based on SIMPER (similarity percentage) analysis of bacterial taxa operational taxonomic units (OTUs), while infants with higher abundance of fecal *Bacteroides* were non-eczemic (Fig. 1A). Representative fecal samples from infants with and without eczema were selected for transplant into mice based on 16S rRNA gene sequencing (Fig. 1B), canonical correspondence analysis (CCA), and SIMPER results. Four samples with elevated *Enterobacteriaceae* that were also closely grouped in the CCA were selected and designated as Infant A microbiota (Fig. 1B). All had high levels of *Escherichia*/*Shigella* and low levels of *Bacteroides* 16S sequence reads, and donor infants had eczema diagnosed at four or five time points between 3 and 36 months of age. Three fecal samples with high levels of *Bacteroides* reads from infants with no eczema or other allergic responses were selected and designated as Infant B microbiota (Fig. 1B).

### Infant A and B microbiotas stabilized, adapted to mouse diet, and passed vertically to offspring

**Fig 2:**
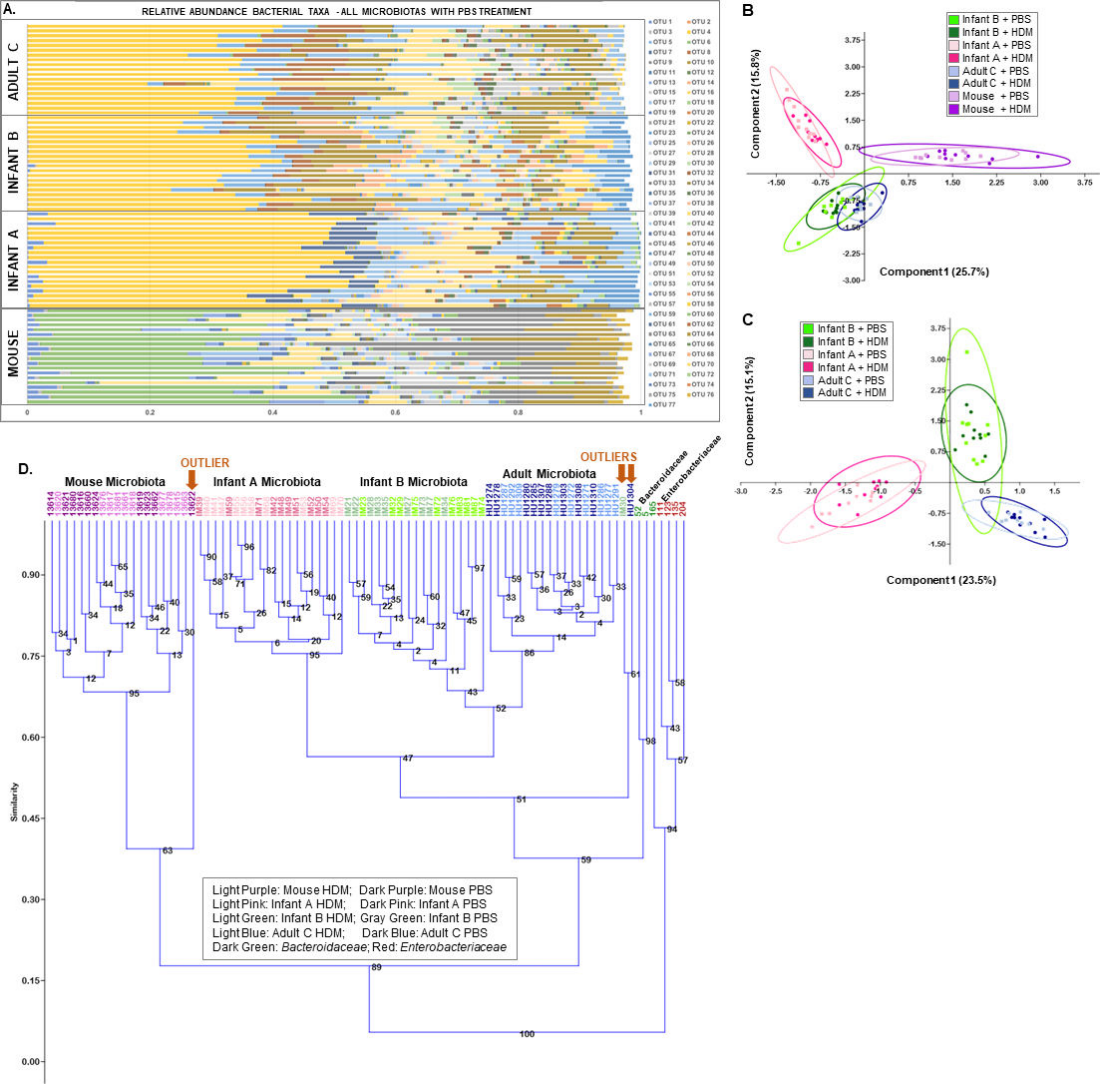
Comparisons of 16S rRNA gene sequencing of all mouse groups. (**A**) Shows relative taxa abundance of mice in all experimental groups, showing Adult C, Infant B, Infant A, and Mouse microbiotas. (**B**) Principal component analysis (PCA) of Infant A, Infant B, Adult C, and Mouse microbiota bacterial taxa abundance. (**C**) PCA of Infant A, Infant B, and Adult C human-derived microbiota bacterial taxa abundance. (**D**) UPGMA clustering with Bray-Curtis similarity index with 1,000 bootstrap replications of all mouse samples and *Enterobacteriaceae-* or *Bacteroidaceae*-dominant inocula showing clustering by microbiota groups. Treatment of mice with phosphate-buffered saline (PBS) or house dust mite (HDM) did not produce recognizable differences in fecal bacterial taxa. Outliers are shown with arrows.

**Fig 3:**
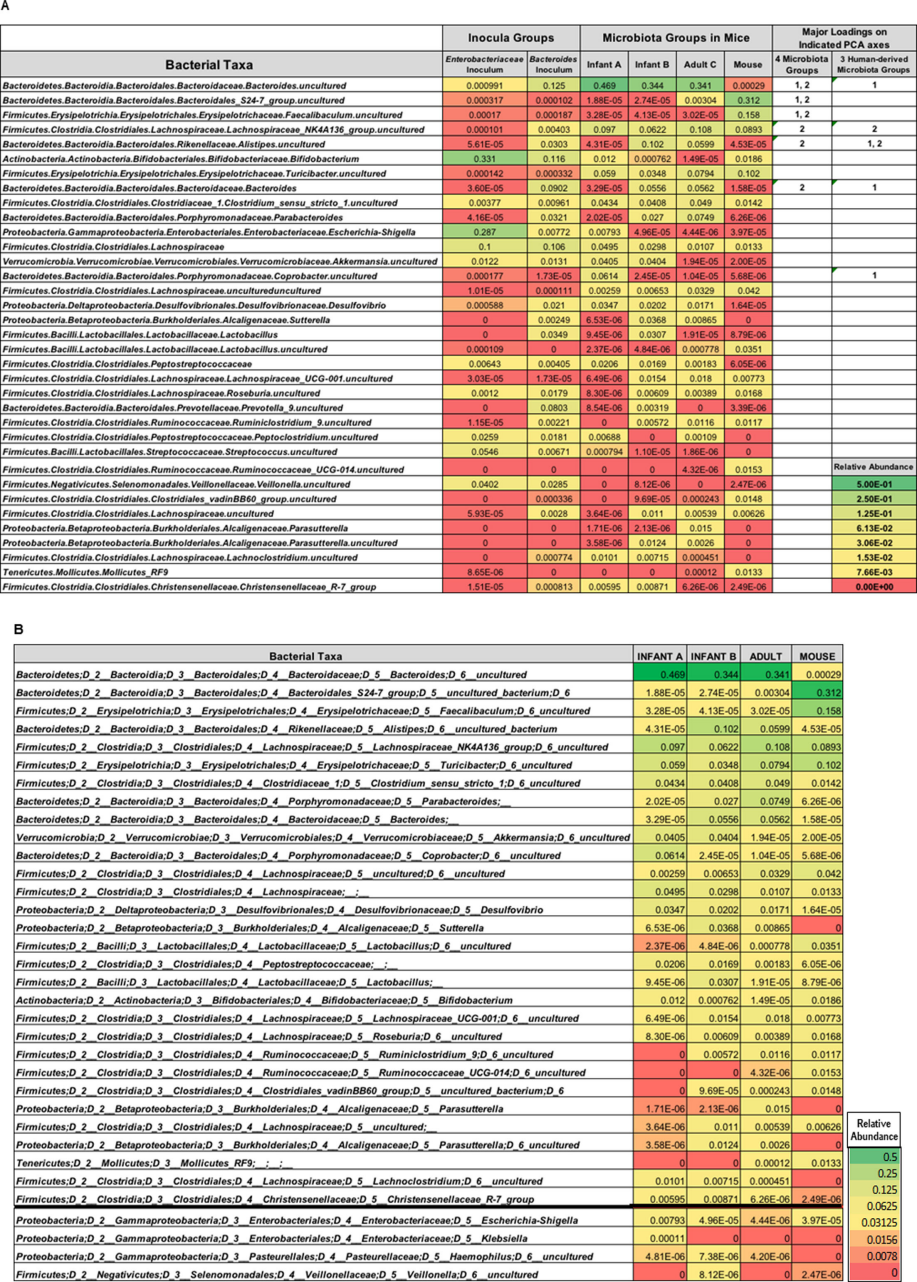
Average relative abundance of all bacterial taxa. (**A**) Heatmap of taxa collectively contributing 90% of differences in SIMPER analysis and having high loadings in the principal component analysis for the inocula (*Enterobacteriaceae* enriched, *Bacteroidaceae* enriched), the human-derived microbiota (Infant A, Infant B, and Adult C), and Mouse microbiota. (**B**) SIMPER analysis of 90% of cumulative bacterial taxa from mice transplanted with Infant A, Infant B, and Adult C human-derived fecal microbiota compared to Mouse microbiota. Top of table shows taxa collectively contributing 90% of difference in SIMPER; taxa contributing <90% of difference are shown below the black line.

Mixed fecal slurries from 3-month-old IOW infants with persistent eczema, “Infant A,” and without eczema, “Infant B,” were transplanted into germ-free mice via oral gavage. First- and second-generation offspring were used for two experiments to test effects of these microbiotas on enhancing allergic airway disease after HDM exposure. Individual mouse-to-mouse variation in human-derived fecal communities was minimal (Fig. 2A). ^MO^Microbiotas were only slightly more variable (Fig. 2A). Principal component analysis (PCA) showed all four microbiotas were distinct (Fig. 2B). The major taxa contributing to separation in dimension 1 of the PCA of all four microbiotas included *Bacteroides* uncultured, *Bacteroidales* S24.7 uncultured, and *Faecalibacterium,* while those contributing to dimension 2 were *Bacteroides uncultured, Lachnospiraceae, Bacteroidales* S24.7, *Faecalibacterium, Alistipes,* and *Bacteroides* (Fig. 2B and 3, last three columns). The major taxa contributing to separation in dimension 1 of the PCA of the three transplanted microbiotas included *Bacteroides* uncultured, *Alistipes*, *Bacteroides,* and *Coprobacter,* while those contributing to dimension 2 were *Lachnospiraceae* and *Alistipes* (Fig. 2C). Unweighted pair groups with arithmetic averaging (UPGMA) dendrogram analysis in experiment 1 showed experimental mice with the same microbiota given either phosphate-buffered saline (PBS) or HDM (Fig. 2D) grouped together posttreatment; HDM did not cause large shifts in microbiotas.

### Human-derived taxa passed to offspring largely unchanged but underwent abundance shifts and a few losses

SIMPER showed contributions of each taxon to observed similarity (or dissimilarity) between samples (Fig. 3A and B). High-abundance taxa in Infant A inoculum, *Bifidobacterium, E. coli-Shigella, Lachnospiraceae, Roseburia*, *Peptoclostridium,* and *Streptococcus,* transferred to mice and decreased in abundance, while *Clostridium sensu stricto*, *Akkermansia*, *Desulfovibrio*, *Peptostreptococcaceae,* and *Veillonella* transferred and increased in abundance. Infant A mice lost *Ruminiclostridium* and *Mollicutes* taxa, which were very low in abundance in the inoculum. High-abundance taxa in Infant B inoculum, *Bifidobacterium, Bacteroides*, *E. coli-Shigella, Lachnospiraceae, Prevotella*, *Peptoclostridium,* and *Streptococcus,* transferred to mice and decreased in abundance, while uncultured *Bacteroides, Lachnospiraceae* NK4A136*, Alistipes*, *Clostridium sensu stricto, Akkermansia*, *Sutterella*, *Peptostreptococcaceae*, uncultured *Lachnospiraceae*, *Lachnoclostridium,* and *Christensenellaceae* R-7 transferred and increased in abundance. Other Infant A or B taxa transferred to mice but stayed at similar abundances.

### Transplanted infant communities retained diversity of donor samples and had distinct compositions

**Fig 4:**
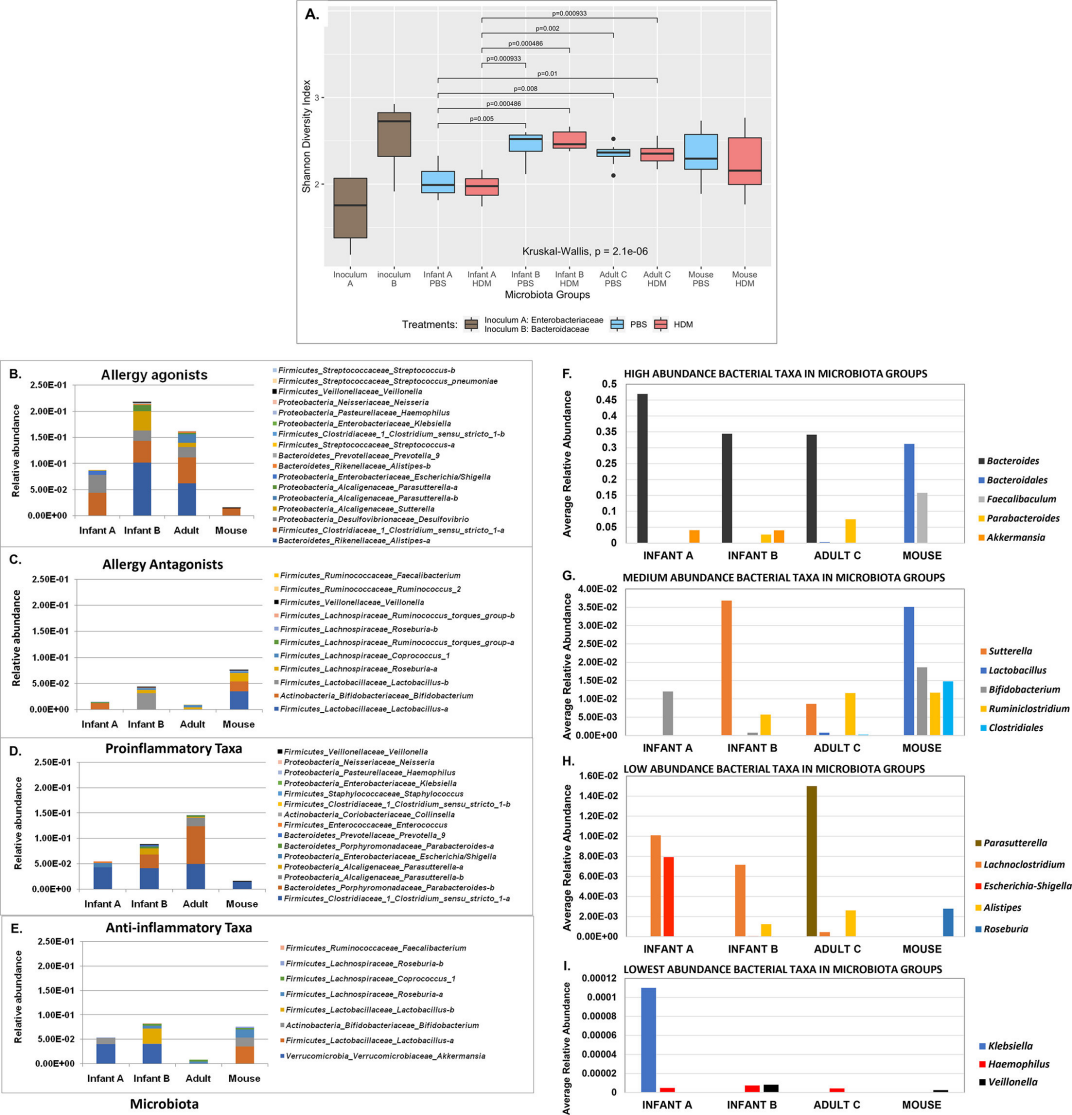
Characteristics of bacterial taxa in mouse models. (**A**) Shannon alpha diversity index performed on operational taxonomic units from *Enterobacteriaceae* inoculum, *Bacteroidaceae* inoculum, Infant A mice given phosphate-buffered saline and house dust mite, Infant B mice given phosphate-buffered saline and house dust mite, Adult C mice given phosphate-buffered saline and house dust mite, and conventional Mouse microbiota mice given phosphate-buffered saline and house dust mite. *Enterobacteriaceae* inoculum and Infant A mice given phosphate-buffered saline and house dust mite had significantly lower diversity than all the other groups. (**B**) Allergy agonists were abundant in human-derived microbiotas compared to Mouse microbiotas, while (**C**) allergy antagonists were numerous in Mouse microbiotas and low in human-derived microbiotas. (**D**) Pro-inflammatory taxa were abundant in human-derived microbiotas and low or absent in Mouse microbiotas, while (**E**) anti-inflammatory taxa were abundant in Mouse microbiotas and few in human-derived microbiotas. (F through I) Show average relative abundance of bacterial taxa grouped according to the four microbiotas Infant A, Infant B, Adult C, and Mouse. The four charts are grouped by abundance as (**F**) high abundance, (**G**) medium abundance, (**H**) low abundance, and (**I**) lowest abundance. Each graph shows the taxa displayed in that chart (see keys). Note that many taxa are found only in the human-derived or the Mouse microbiota.

Bacterial taxa present in three ^HU^microbiotas but absent in ^MO^microbiota included *Akkermansia*, *Parasutterella*, *Peptostreptococcaceae, Lachnoclostridium,* and *Sutterella*. An uncultured *Bacteroides* taxon was elevated in all three human-derived microbial communities but absent in mice with ^MO^microbiota. An *Escherichia-Shigella* OTU was abundant in Infant A, present at very low levels in Infant B, and undetectable in Adult C microbiotas. *Bifidobacterium* and *Akkermansia* were more abundant in Infant A than in Infant B mice. *Coprobacter* was in Infant A but not in Infant B mice. *Alistipes*, *Parabacteroides*, *Lactobacillus*, *Parasutterella* uncultured, *Sutterella*, *Lachnospiraceae*, *Roseburia*, *Ruminiclostridium,* and *Blautia* taxa were more abundant in Infant B than in Infant A mice. Infant A mice had *Klebsiella* that was absent in other microbiotas. *Lactobacillus*, *Parasutterella,* and a *Clostridiales* vadinBB60 species were present in Adult C microbiotas but not in either Infant microbiotas. Thus, many bacterial taxa were distinct to each microbiota, and a few were shared between microbiotas. Three of four microbiotas were diverse (Fig. 4A). Shannon alpha diversity analyses were not significantly different between donor Infant A inoculum and Infant A mice, and between donor Infant B inoculum and Infant B mice (Fig. 4A). Infant A donor inoculum and Infant A Mouse microbiota were significantly decreased in diversity compared to Infant B inoculum, Infant B Mouse microbiota, Adult C Mouse microbiota, and control ^MO^microbiota (Fig. 4A).

### Allergy agonists/antagonists and pro-/anti-inflammatory taxa varied in abundance between ^HU^microbiotas versus ^MO^microbiotas

We compared the average relative abundance of bacterial taxa known to exacerbate allergy as allergy agonists or protect against allergy as antagonists, and those known to be pro- or anti-inflammatory in ^HU^microbiotas versus ^MO^microbiotas (Fig. 4B through E). The total fraction of allergy agonists was greater in ^HU^microbiotas than in ^MO^microbiotas (Fig. 4B; Table 1); greater numbers of individual agonist taxa than antagonist taxa were detected. The situation in ^MO^microbiota was reversed–allergy agonist taxa were in smaller proportions and fewer in number of individual taxa, while allergy antagonist taxa were in higher proportions with more individual taxa (Fig. 4B; Table 1).

**TABLE 1:** Bacterial taxa found in Infant A, Infant B, Adult C, and Mouse microbiotas known to act as allergy agonists or antagonists based on published studies (15, 22, 25–27, 29–35)

Allergy agonist or antagonist	Infant A	Infant B	Adult C	Mouse
Agonists				
*Streptococcus pneumoniae*	1	1	1	0
*Moraxella catarrhalis*	0	0	0	0
*Neisseria*	0	0	0	0
*Veillonella*	0	1	0	0
*Clostridium* cluster I	1	1	1	1
*Desulfovibrio*	1	1	1	0
*Sutterella*	0	1	1	0
*Parasutterella*	0	0	1	0
*Escherichia coli/Shigella*	1	0	0	0
*Klebsiella*	1	0	0	0
*Haemophilus*	1	1	1	0
*Prevotella*	0	1	0	0
*Alistipes*	0	1	1	0
Total agonist taxa	6	8	7	1
Antagonists				
*Clostridia* (*Clostridiales*)	0	0	0	1
*Lachnospira*	1	1	1	1
*Veillonella*	1	0	0	0
*Faecalibacterium*	0	0	0	1
*Rothia mucilaginosa*	0	0	0	0
*Roseburia*	0	1	1	1
*Ruminococcus, Coprococcus*	0	0	0	1
*Lactobacillus*	0	1	0	1
*Bifidobacteria*	1	0	0	1
Total antagonist taxa	3	3	2	6

More individual pro-inflammatory taxa were detected than anti-inflammatory taxa in this data set (Fig. 4D and E). ^HU^Microbiotas had more pro-inflammatory taxa than ^MO^microbiotas, and each ^HU^microbiota had particular pro-inflammatory taxa present or in greater abundance (Fig. 4D; Table 2). Only *Bacteroides* and *Haemophilus* were seen in all ^HU^microbiotas (Fig. 4F and I). Infant A mice had more *E. coli-Shigella* (Fig. 4H). Infant B and Adult C had one pro-inflammatory taxon in common, *Parabacteroides* OTU59 (Fig. 4F). Adult C had higher levels of *Parasutterella* OTUs (Fig. 4H). Adult C microbiota mice had the largest abundance of pro-inflammatory taxa (Fig. 4D; Table 2). Outcomes in ^MO^microbiota composition of pro- and anti-inflammatory taxa were reversed (Fig. 4D and E). While all microbiotas had some anti-inflammatory taxa, ^MO^microbiota had more genera that were more abundant (Fig. 4E; Table 2). Known anti-inflammatory taxa present in ^MO^microbiotas and absent or at exquisitely low amounts in ^HU^microbiotas were *Bacteroidales*, *Faecalibacterium*, *Lactobacillus*, *Roseburia, Clostridiales* , and *Ruminococcaceae* (Fig. 4F through H).

**TABLE 2:** Bacterial taxa found in Infant A, Infant B, Adult C, and Mouse microbiotas known to function in a pro-inflammatory or anti-inflammatory manner in acute or chronic diseases based on published studies (15, 22, 25–27, 29–35)^*a*^

Taxon	Infant A	Infant B	Adult C	Mouse
Pro-inflammatory taxa				
*Clostridia/C. perfringens/C. difficile*	1	1	1	1
*Collinsella*	0	1	1	0
*Enterobacter aerogenes*	0	0	0	0
*Enterococci*	1	0	0	0
*Escherichia coli/Shigella* OTU42	1	1	0	0
*Haemophilus*	1	1	1	0
*Klebsiella*	1	0	0	0
*Moraxella*	0	0	0	0
*Morganella morganii*	0	0	0	0
*Neisseria*	0	0	0	0
*Parabacteroides* OTU59	0	1	1	0
*Parasutterella* OTU26	0	1	1	0
*Prevotella*	0	1	0	0
*Staphylococcus aureus*	0	0	0	0
*Veillonella*	0	1	0	0
Total pro-inflammatory taxa	5	8	5	1
Anti-inflammatory taxa				
*Akkermansia muciniphila*	1	1	0	0
*Bacteroides*	1	1	1	0
*Bifidobacterium* OTU18	1	0	0	1
*Coprococcus eutactus*	0	1	0	1
*Faecalibacterium prausnitzii*	0	0	0	0
*Lactobacillus*	0	0	0	1
*Lactobacillus uncultured* OTU7	0	0	0	1
*Roseburia* OTU	0	1	0	1
*Roseburia* OTU	0	0	0	1
Total anti-inflammatory taxa	3	4	1	6

^*a*^ Data represent the presence/absence values. Microbiotas were scored as 1 if a particular taxon was present and 0 if it was not present.

### Effects of microbiota on baseline pulmonary mechanics (experiment 1)

**Fig 5:**
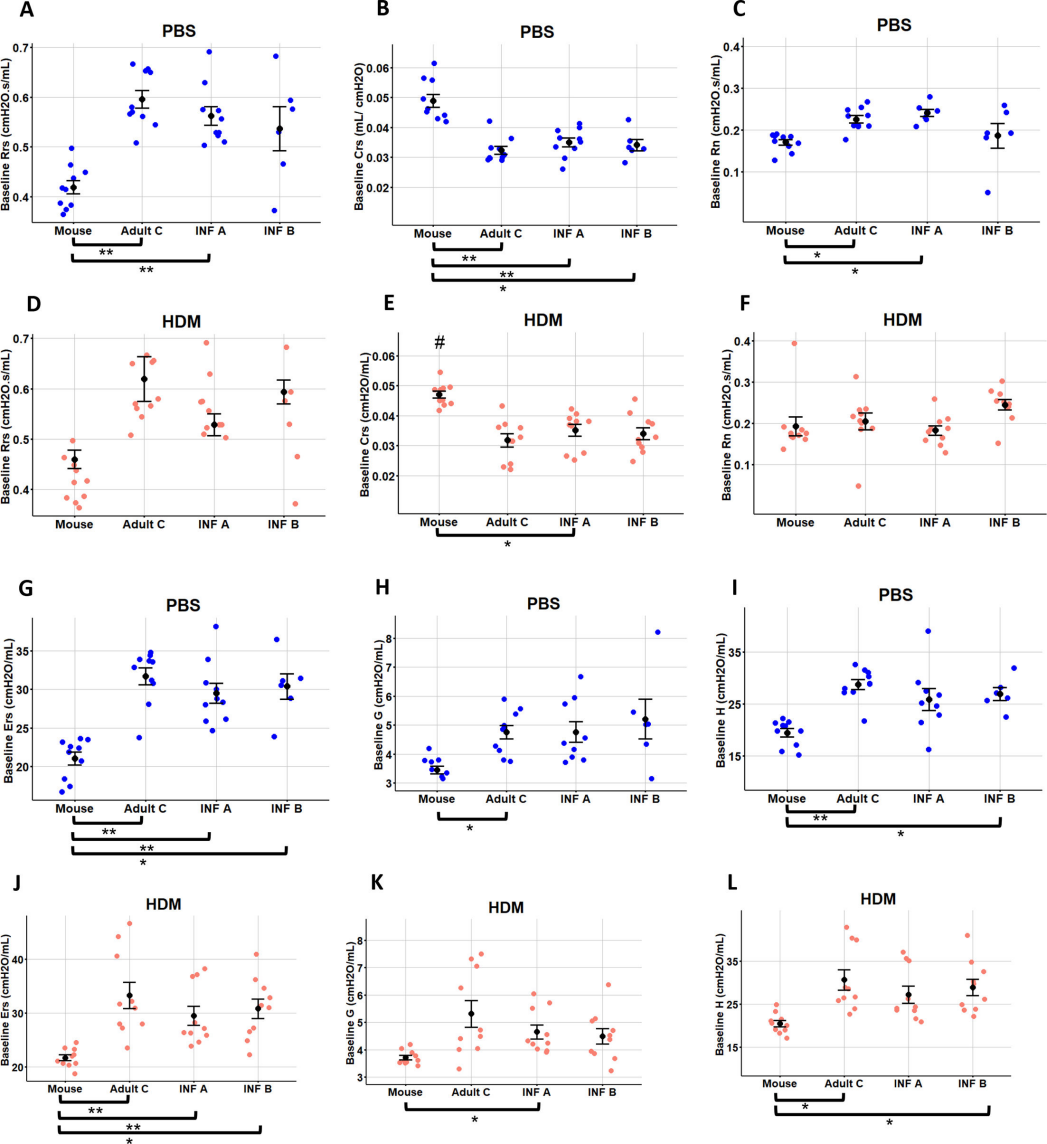
Lung baseline mechanics for mice with all three human microbiotas were significantly different from the controls carrying Mouse microbiota. Measurements at peak responses of resistance (Rrs in cmH2O.s/mL), compliance (Crs in mL/cmH2O), elastance (Ers in cmH2O/mL), tissue elastance (H in cmH2O/mL), tissue damping (G in cmH2O/mL), and conducting airway (Newtonian) resistance (Rn in cmH2O.s/mL) for each methacholine dose for PBS and HDM treatment groups produced usable data for all mice tested to assess airway hyperresponsiveness. Data show examination of baseline lung mechanics for (**A, D**) total airway resistance Rrs, (**B, E**) total airway compliance Crs, (**C, F**) central airway resistance Rn, (**G, J**) tissue elastance Ers, (H, K) peripheral airways and tissue damping G, and (**I, L**) tissue elastance H for mice treated with PBS or HDM. Data represent the mean ± SE from 10 mice per group. Data analyzed with Kruskal-Wallis with Mann-Whitney for pairwise comparisons. *P < 0.05, **P < 0.01, #*P* < 0.05 compared to the same microbiota with different treatments.

**Fig 6:**
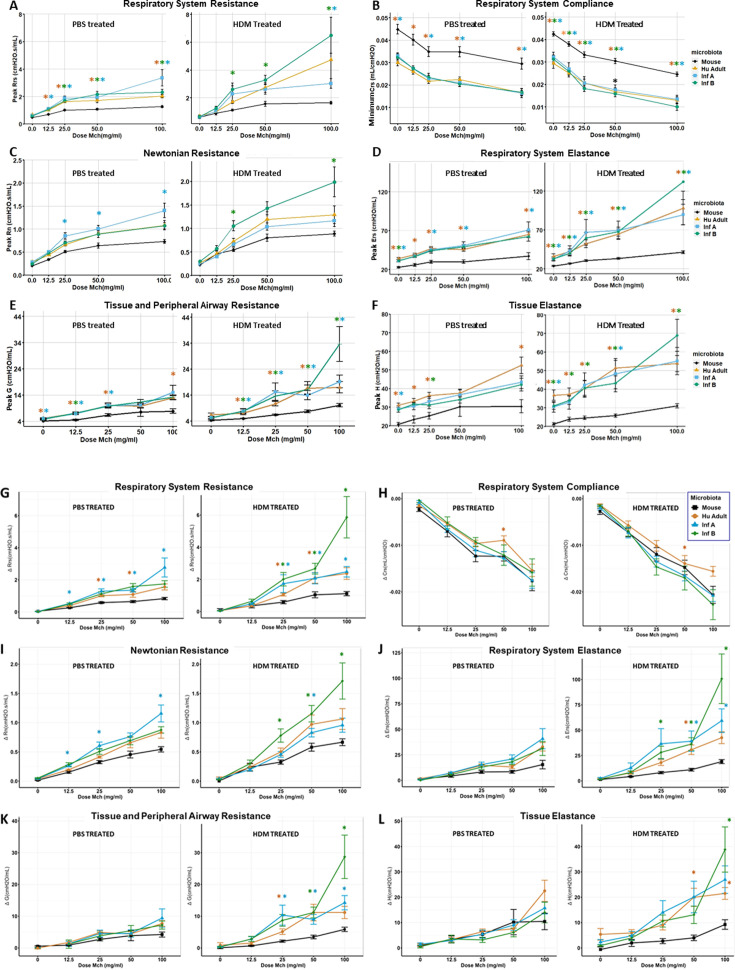
Lung functions based on flexiVent baseline values showed that mice with human-derived microbiotas had respiratory impairment. (A–F) Data show peak or minimum lung function value averages ± standard error of the mean analyses performed on the top or the bottom three values using Kruskal-Wallis and Mann-Whitney with Bonferroni corrections. Significant comparisons *P* < 0.05 are shown as *asterisk shown in color of microbiota group. When given either PBS or HDM, ^HU^microbiota mice had elevated dose–response curves for peak Rrs, peak Rn, Ers, G, and H values based on flexiVent assessments compared to ^MO^microbiota controls (A, C–F). They also had decreased dose–response curves for airway compliance (Crs) when compared to ^MO^microbiota controls (B). All responses were more pronounced in allergic (HDM) conditions. (G–L) Lung functions based on flexiVent values adjusted to remove baseline responses showed that mice with human-derived microbiotas had respiratory impairment. By comparing graphs in panels A–F with those in panels G–L, it is clear that respiratory system compliance (Crs), tissue and peripheral airway resistance (**G**), and tissue elastance (**H**) are mainly mediated by microbiota effects on baseline responses.

C57BL/6 mice were used because their modest response to HDM allows detection of (microbiota + HDM) responses. All mouse groups were given 30–50 μg HDM or PBS intranasally for 2 weeks (Table 3). On day 14, respiratory system resistance (Rrs), compliance (Crs), elastance (Ers), Newtonian resistance (Rn), tissue and peripheral airway resistance (G), and tissue elastance (H) were measured using flexiVent to assess lung function. Lung mechanics are shown first using baseline values from the flexiVent before MCh is given (Fig. 5). At baseline, all ^HU^microbiota mice had a significantly different lung function under nonallergic (PBS) and allergic (HDM) conditions than ^MO^microbiota mice (Fig. 5). Rrs—that has contributions from conducting and peripheral airways, lung tissue, and chest wall—was increased in ^HU^microbiota mice with significance in Adult C and Infant A (Fig. 5A). Respiratory system compliance (Crs)—the ease with which the respiratory system can be expanded—was significantly decreased in all three ^HU^microbiota mice given PBS (Fig. 5B). Respiratory system elastance (Ers)—the elastic stiffness of the respiratory system—was significantly increased in ^HU^microbiota mice compared to ^MO^microbiota controls given PBS or HDM (Fig. 5G and J). ^HU^Microbiota mice had higher values for peripheral airway resistance (G) than ^MO^microbiota mice with significance for Adult C mice with PBS (Fig. 5H) and Infant A mice with HDM (Fig. 5K). Infant B and Adult C ^HU^microbiota mice had significantly higher tissue elastance (H) responses than ^MO^microbiota mice in HDM and PBS conditions (Fig. 5I and L). Central airway constriction (Rn) was significantly increased in Infant A and Adult C microbiota mice compared to ^MO^microbiota mice under PBS conditions (Fig. 5C). Thus, mice carrying any ^HU^microbiota had different lung mechanics at baseline with greater lung stiffness, airway resistance, and decreased compliance compared to control mice. No significant differences were found in lung function between PBS- or HDM-treated mice carrying different ^HU^microbiotas when compared to each other (PBS,Fig. 5A through C and G through I; HDM, Fig. 5D through F and J through L). Thus, ^HU^microbiotas impaired baseline lung function. Relative total proportions of allergy agonist and pro-inflammatory taxa match trends of higher severity of lung function impairment in ^HU^microbiota mice (Fig. 5 and 6; Table S2).

**TABLE 3:** Design for experiment 1 shows the four gut microbiotas used and the number (N) of male (M) and female (F) mice in each group of microbiota and treatment combination^*b*^

Microbiota	Treatment	N and sex	Treatments administered^*a*^						Phenotype
			Day 0	Day 2	Day 5	Day 7	Day 9	Day 12	
Mouse control	PBS	5M, 5F	25 μL	30 μL	30 μL	30 μL	30 μL	30 μL	Day 14
Mouse control	HDM	5M, 5F	50 μg	30 μg	30 μg	30 μg	30 μg	30 μg	Day 14
Infant A	PBS	5M, 5F	25 μL	30 μL	30 μL	30 μL	30 μL	30 μL	Day 14
Infant A	HDM	5M, 5F	50 μg	30 μg	30 μg	30 μg	30 μg	30 μg	Day 14
Infant B	PBS	3M, 3F	25 μL	30 μL	30 μL	30 μL	30 μL	30 μL	Day 14
Infant B	HDM	5M, 5F	50 μg	30 μg	30 μg	30 μg	30 μg	30 μg	Day 14
Adult C	PBS	5M, 5F	25 μL	30 μL	30 μL	30 μL	30 μL	30 μL	Day 14
Adult C	HDM	5M, 5F	50 μg	30 μg	30 μg	30 μg	30 μg	30 μg	Day 14

^*a*^ All HDM extract administered in 0.2 mL volume of PBS.

^*b*^ Each group was treated with phosphate-buffered saline (PBS) control or house dust mite (HDM) extract intranasally on days 0, 2, 5, 7, 9, and 12. On day 14, several phenotypes were assessed. Specifically, lung function measurements were taken using flexiVent over the course of increasing doses of aerosolized methacholine. Thereafter, bronchoalveolar lavage, blood, lung, and gastrointestinal samples were collected. The design for experiment 2 was exactly the same as in experiment 1, except that we used only mice carrying Infant B or Infant A microbiota, and mice with Adult C and Mouse microbiota were not used.

### Effects of microbiota on MCh-driven pulmonary mechanics (experiment 1)

After recording baseline lung mechanics in experiment 1, the muscarinic agonist MCh was given at increasing doses via nebulization to evaluate AHR in mice with (allergic) and without (nonallergic) HDM. MCh acts directly on muscarinic receptors of airway smooth muscle inducing bronchoconstriction, which is a sensitive tool to confirm/exclude asthma (41). When given either PBS or HDM, ^HU^microbiota mice had elevated dose–response curves for peak Rrs, peak Rn, Ers, G, and H values based on flexiVent assessments compared to ^MO^microbiota controls (Fig. 6A, C through F). They also exhibited dose–response curves indicating decreases in airway compliance (Crs) when compared to ^MO^microbiota controls (Fig. 6B). All responses were more pronounced in allergic (HDM) conditions. When flexiVent values were adjusted to remove baseline, mice with ^HU^microbiotas had the greatest impairment in peak Rrs, peak Rn, Ers, G, and H compared to conventional Mouse microbiota mice, mainly under allergic conditions (Fig. 6G through L). Despite considerable variability in the data, conventional microbiota mice showed statistically significant differences from ^HU^microbiota mice in lung parameters Rrs, Ers, Rn, and G at multiple MCh doses (Fig. 6G through L; Table S2).

**Fig 7:**
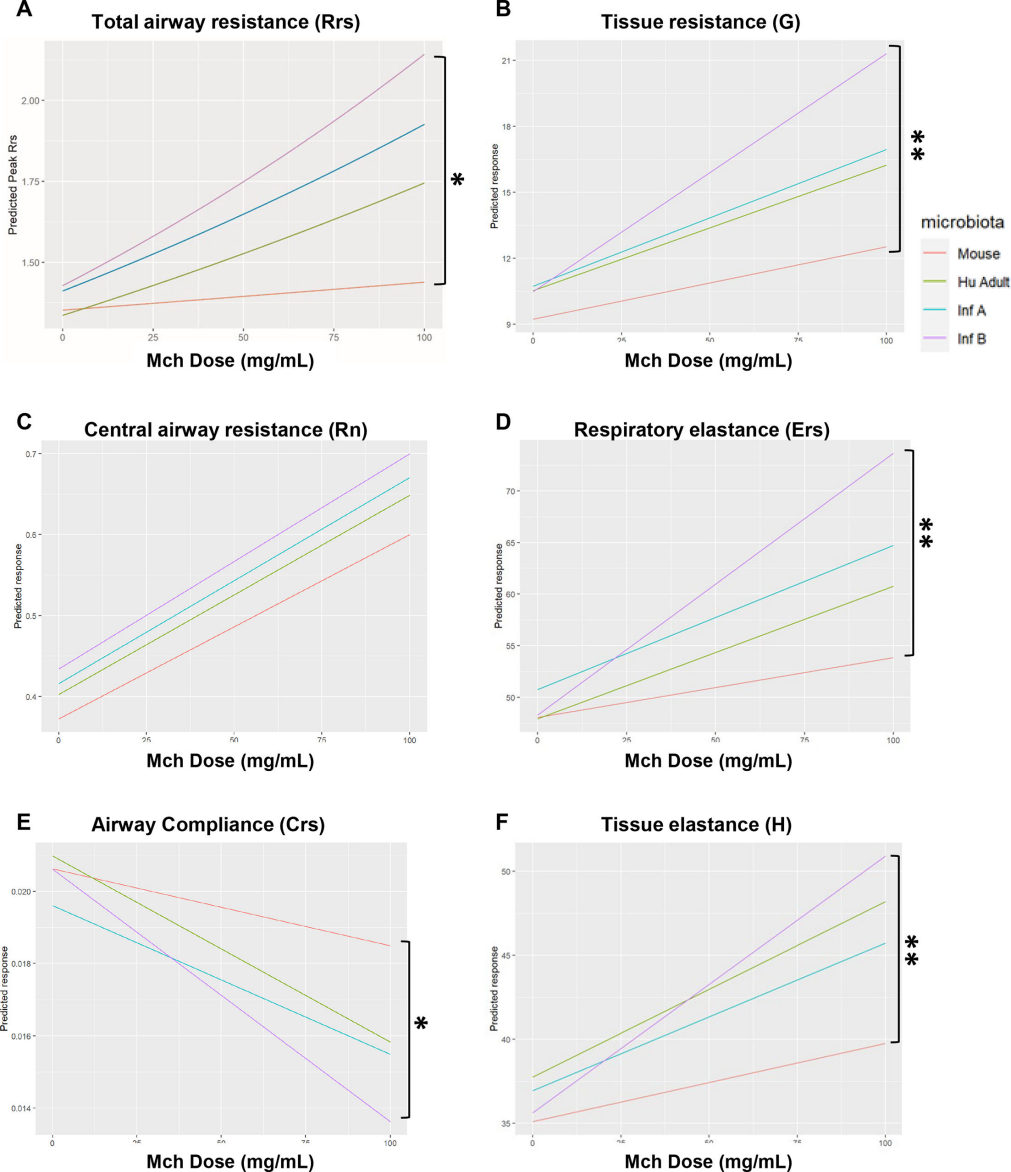
General linear mixed model. Mice carrying Infant B microbiota showed increased airway hyperresponsiveness after methacholine (MCh) challenge compared to mice with control microbiota. Lines represent predicted change in peak value of lung function for each unit increase of MCh using a general linear mixed (GLM) model for the corresponding microbiota. Data represented on each line include mice from both PBS and HDM treatment groups for (**A**) total airway resistance, (**B**) tissue resistance, (**C**) central airway resistance, (**D**) respiratory elastance, (**E**) airway compliance, and (**F**) tissue elastance. Pairwise comparisons were used to compare slopes. *P < 0.05, **P < 0.01.

To further explore differences observed at baseline between mice carrying different microbiotas, a general linear mixed model (GLMM) was used to confirm the observed dose–response data and generate predicted dose–response curves (data in Tables S2 and S3). Based on GLMM, the effect of an increase in dose of MCh (levels = 0, 12.5, 25, 50, 100 mg/mL) on Rrs was significant for all microbiota groups ($\chi^{2}=\;9.1195,\;P=0.0277$) (Fig. 7A). Differences in resistance between microbiotas were mainly due to increased total Rrs and tissue resistance (G) in smaller airways (Fig. 7B), while no significant differences were seen on resistance of larger airways between microbiota groups ($\chi^{2}=2.1436\;,\;P=0.5432$) (Fig. 7C). AHR in mice carrying ^HU^microbiotas was characterized by an overall increased respiratory system elastance (Ers) (Fig. 7D) and a decrease in its reciprocal, lung compliance (Crs) (Fig. 6E). Although all ^HU^microbiotas affected these lung functions, Infant B had the greatest effects (Table S4). For both PBS and HDM conditions, no differences were found between male and female baseline values for any lung parameters measured, although this study was not designed or powered to determine the effect of sex on AHR.

### HDM elevated lung inflammation in mice of all microbiotas, but certain cell types and cytokines (IL-5, IL-13) varied based on microbiota

**Fig 8:**
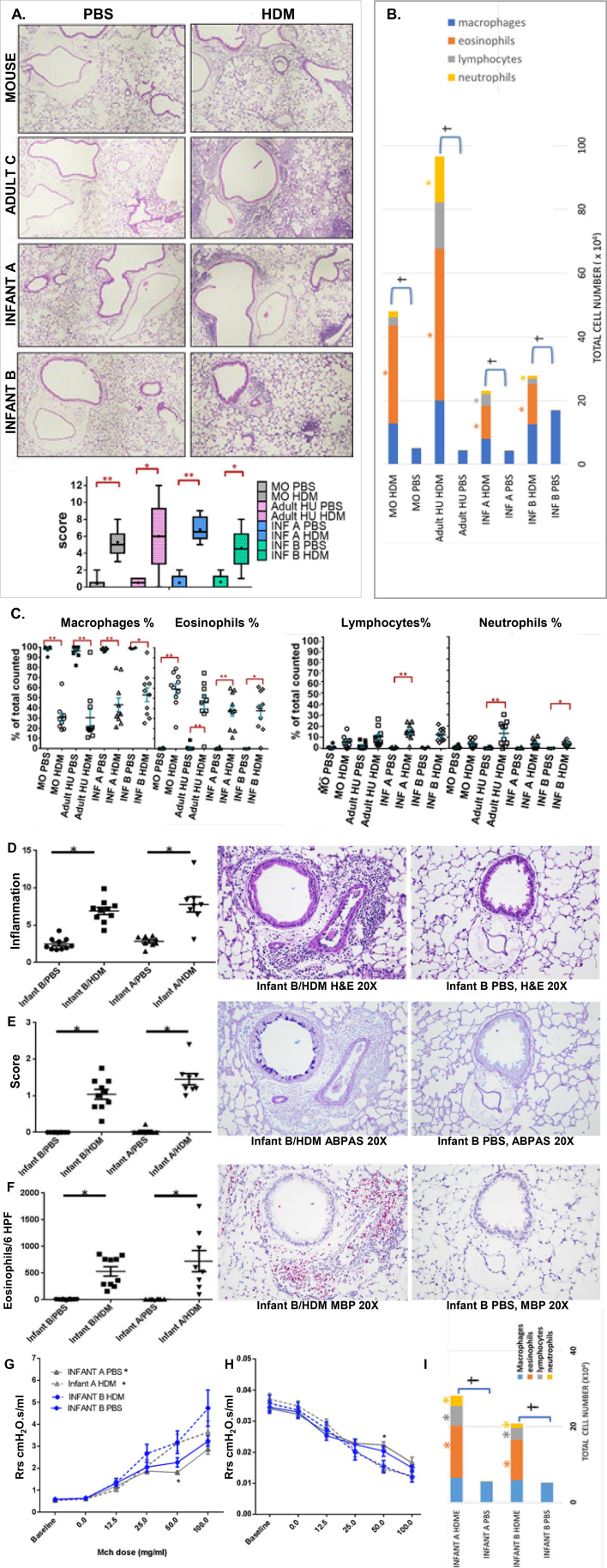
HDM increased lung tissue inflammation for all four types of microbiotas. (**A**) Lung histology and scores for each group. Representative images from each experimental group of lung tissue show the presence or absence of inflammation surrounding large and smaller airways as well as blood vessels. (**B**) Total cell numbers from bronchoalveolar lavage with differential cell counts. Differential cell counts were made on 300 total cells counted per slide. (**C**) Percentages of each cell type out of the total cell count in the bronchoalveolar lavage as assessed by the differential cell count. Data represent the mean ± SE. *P < 0.05, **P < 0.01. Quantification of (**D**) inflammation in hematoxylin and eosin-stained lung sections, (**E**) mucus cell metaplasia in Alcian blue PAS-stained lung sections, and (**F**) density of eosinophils on major basic protein stained lung sections. For all three stains, a significant difference (*P* < 0.05) was observed between groups that were HDM^+^ compared to those that were HDM^–^. No significant difference was observed between groups of the same HDM condition. Statistical analysis was performed as a one-way ANOVA with Kruskal-Wallis, followed by Dunn’s post hoc test, or with Holmes-Sidak, depending on normality. Data represent the mean ± SE from 10 mice per group. Groups that complied with normality were compared using ANOVA with Holmes-Sidak, while those that did not were compared using ANOVA with Kruskal-Wallis, followed by a Dunn’s post hoc test. *P < 0.05, **P < 0.01. (**G**) Total airway compliance and (**H**) total airway resistance of mice at baseline and after receiving increasing doses of MCh. (**I**) Stacked bar graphs represent the total cell number in BAL with proportions for each cell type in different colors based on differential cell count performed in 300 cells per field. * = *P* < 0.05 for specific cell type compared to the same microbiota but with PBS , † = *P* < 0.05 for total cell count compared to the same microbiota but with PBS. For panels **G** and **H**, data represent the mean ± SE. Data analyzed with Kruskal-Wallis with Mann-Whitney for pairwise comparisons. Significant comparisons are shown as *P < 0.05, **P < 0.01. One individual value for panel **E** from group Infant B PBS is not shown as it falls outside of the axis limit (value = 1,425 ng/mL).

Bronchoalveolar lavage fluid (BALF) and lung sections were evaluated for tissue inflammation, airway remodeling, innate cells, mucus cell metaplasia, and cytokines, including Th1, Th2, and Th17 families. HDM increased inflammation in lungs of mice of all microbiotas compared to their PBS controls (Mouse *P* = 0.003, Adult C *P* = 0.033, Infant A *P* = 0.003, Infant B *P* = 0.038), but histopathology scores were highest in Adult C mice (Fig. 8A). Inflammation was concentrated around mid- to small-sized airways in HDM-treated mice (Fig. 8A). BALF inflammatory cell proportions changed in response to HDM in mice of all microbiotas, but Adult C mice had the greatest increases in eosinophils, lymphocytes, and neutrophils (Fig. 8B). Significant increases in BALF relative cell counts (mainly eosinophils) were seen in HDM- versus PBS-treated mice (Mouse *P* = 0.003, Adult C *P* = 0.005, Infant A *P* = 0.004, Infant B *P* = 0.048). Only Adult C and Infant B microbiota mice had significant increases in neutrophils after HDM compared to controls (*P* < 0.01 and *P* < 0.05, respectively) (Fig. 8C). Only Infant A microbiota mice had significantly increased lymphocytes after HDM compared to controls (*P* < 0.01) (Fig. 8C). No significant differences were seen in percentage of total BALF cells between microbiotas of PBS controls or HDM-treated mice.

**Fig 9:**
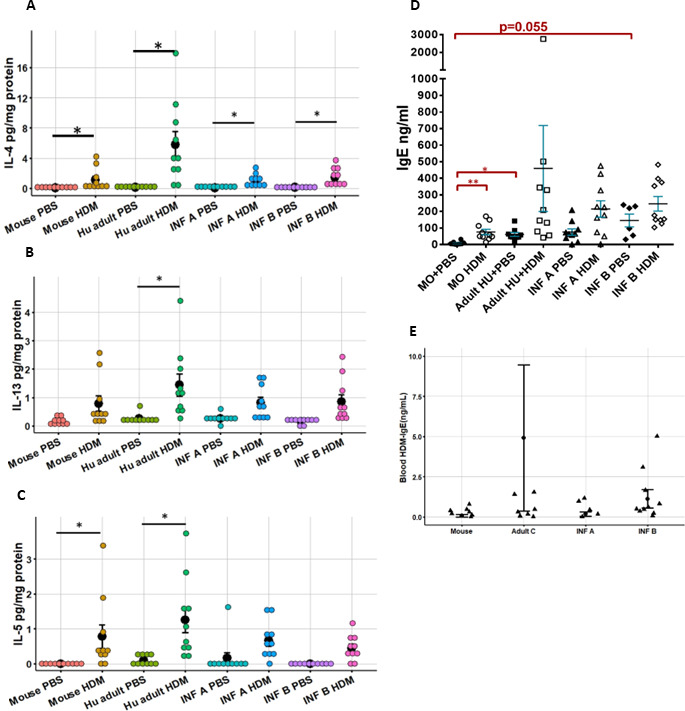
Type 2 immune response to HDM in different mice carrying different microbiotas. (**A**) Interleukin-4 cytokine responses measured in lung tissue. (**B**) Interleukin-13 cytokine responses in lung tissue. (**C**) Interleukin-5 cytokine responses in lung tissue. (**D**) Immunoglobulin E responses in mice with different microbiotas from experiment 1 given phosphate-buffered saline or house dust mite allergen. (**E**) Immunoglobulin E responses in mice with Infant A and Infant B microbiotas from experiment 1 given phosphate-buffered saline or house dust mite allergen. Data represent the mean ± SE from 10 mice per group. Data analyzed with Kruskal-Wallis with Mann-Whitney for pairwise comparisons. *P < 0.05, **P < 0.01.

IL-4 was significantly increased in lung homogenates for HDM- versus PBS-treated mice of mouse (*P* = 0.047), Adult C (*P* = 0.016), Infant A (*P* = 0.019), and Infant B (*P* = 0.035) microbiotas (Fig. 9A). Only mouse and Adult C microbiota mice given HDM had significantly increased IL-5 compared to PBS controls (*P* < 0.05), while only Adult C microbiota given HDM had significantly increased IL-13 compared to PBS controls (*P* < 0.05) (Fig. 9A through C). Other cytokines showed no differences in their levels based on treatment or microbiota type.

### ^HU^Microbiota mice had elevated levels of total serum IgE

**Fig 10:**
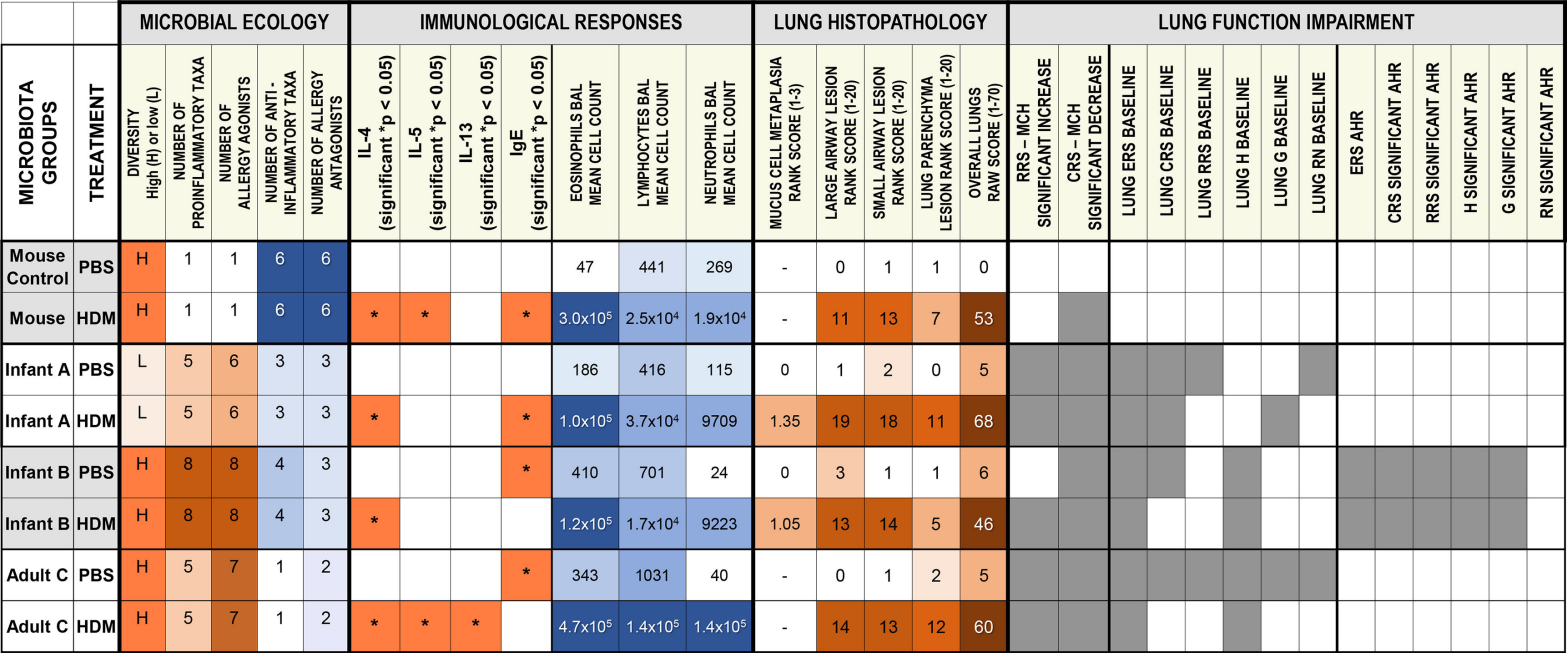
Summary heatmap showing responses of different microbiota groups given phosphate-buffered saline (PBS) or house dust mite (HDM). (**A**) Summary heatmap shows data driving distinctions between lung function impairments in mice carrying one of four microbiota groups given PBS or HDM treatments and assessed using flexiVent, lung bronchoalveolar lavage (BAL) cell counts, lung histopathology scoring, and fecal microbiota16S rRNA gene sequencing. Pro-inflammatory and anti-inflammatory taxa categories and agonist and antagonist categories are ranked; scores for the presence/absence of defined bacteria are shown in Tables 1 and 2. Shannon alpha diversity for the four microbiota groups are shown as high (H) or low (L) diversity. ERS is elastance, CRS is compliance, RRS is respiratory resistance, H is tissue elastance, G is smaller peripheral airways and tissue damping, RN is central airway resistance, and AHR is airway hyperresponsiveness. Methacholine (MCH) administration caused significant increases in lung resistance (RRS) and decreases in compliance (CRS), as shown by gray squares. Significant differences between lung baseline mechanics of groups compared to mice with Mouse microbiota given PBS are also shown by gray squares. Infant B microbiota mice were the only group that had significantly increased airway hyperresponsiveness after MCH challenge when compared to mice with control microbiota given PBS. Bacterial taxa found in Infant A, Infant B, Adult C, and Mouse microbiotas known to act as allergy agonists or antagonists were based on published studies (15, 22, 25–27, 29–35). Bacterial taxa found in Infant A, Infant B, Adult C, and Mouse microbiotas known to function in a pro-inflammatory or anti-inflammatory manner in acute or chronic diseases were based on published studies (15, 22, 25–27, 29–35). Data represent the presence/absence values. Microbiotas were scored as 1 if a particular taxa was present, and 0 if it was not present.

Mice with ^HU^microbiotas had increased levels of serum total IgE compared to mice with ^MO^microbiota in both PBS- and HDM-treated conditions (Fig. 9D). In nonallergic conditions (PBS only), Adult C microbiota (*P* = 0.007) and Infant B microbiota (*P* = 0.055) mice had significantly higher total serum IgE levels compared to ^MO^microbiota controls. Infant B microbiota mice given HDM trended to higher levels of IgE compared to ^MO^microbiota mice given PBS (*P* values = 0.062). Only ^MO^microbiota mice had significantly increased total serum IgE after treatment with HDM compared to PBS controls (*P* = 0.009) (Fig. 9D). Total serum IgE does not reflect a specific HDM-IgE increase because we only detected a few mice with HDM-specific IgE 2 weeks after HDM treatment (Fig. 9E); other studies also show that HDM-specific serum IgE requires more than 2 weeks of HDM treatment to develop (42). Yet, data suggest an association of increased total serum IgE with increased AHR in ^HU^microbiota mice especially in mice under nonallergic conditions with Infant B or Adult C microbiotas (Fig. 10).

### Functional impairment of respiratory system resistance and elasticity in ^HU^microbiota mice was associated with increased total serum IgE but not HDM-specific IgE

A summary heatmap shows data driving distinctions between lung function impairments in mice with ^HU-^ or ^MO^microbiota given PBS or HDM treatments and assessed using flexiVent, lung BALF cell counts, lung histopathology scoring, and microbiota 16S sequencing (Fig. 10). To determine whether there was an association between eosinophilic infiltrates, lung inflammation, and Th17 and Th2 cytokines with lung function, we evaluated the correlation between the presence of these factors and lung function measurements. A statistically significant, moderate to strong positive correlation between overall baseline values for respiratory system resistance and total serum IgE levels was found in all microbiota groups given HDM; the Pearson correlation coefficient for HDM-treated animals was 0.650. Similar results were found for respiratory system compliance (Crs), respiratory system elastance (Ers), tissue and peripheral airway resistance (G), and tissue elastance (H). When evaluating correlations grouped by microbiota, Adult C mice given HDM had a significant strong correlation between Rrs and total IgE at baseline, dose 0 mg/mL of MCh, dose 12.5 mg/mL of MCh, and dose 50 mg/mL, and a significant strong correlation between G and total IgE at dose 12.5 mg/mL of MCh. PBS-treated Infant A microbiota mice had significant strong correlations between several lung function parameters at both baseline and dose 0 mg/mL of MCh challenge. ^MO^Microbiota had a significant strong correlation between Rrs at baseline and IgE but only in PBS conditions. Infant B microbiota had no significant correlations between lung function parameters and total IgE. None of the other variables measured showed a significant correlation with lung function, or with each other, when analyzed by Pearson and Spearman correlation (Table S5).

### Data were repeatable, and Infant B microbiota again acted as an allergy agonist

To confirm previous findings that Infant A and Infant B microbiota mice produced similar AHR, we conducted a second experiment with four groups, (i) Infant A with PBS, (ii) Infant A with HDM, (iii) Infant B with PBS, and (iv) Infant B with HDM. Mice were sensitized to PBS or HDM for 14 days and phenotyped for lung function. No significant differences in lung function at baseline were seen between Infant A or B microbiota mice, given the same treatments. We did not observe a significant difference in responses to increasing doses of MCh or AHR, measured as total airway resistance and compliance (Fig. 8G and H). Furthermore, baseline lung function values and values taken at different doses of MCh for each microbiota were similar between experiment 1 and experiment 2 for mice in the same treatment group with the same microbiota (data not shown). As in experiment 1, there were no significant differences between AHR responses between mice with the same microbiota treated with HDM and PBS, except for Infant A microbiota at dose 50 mg/mL of MCh, in which the increase in airway resistance and decrease in airway compliance were significantly higher than the PBS-treated group (Fig. 8G and H). Overall similar results from experiments 1 and 2 support rejection of our hypothesis that Infant B microbiota decreased allergic response compared to Infant A microbiota (Table 4).

**TABLE 4:** Comparison of average ± SEM values obtained from total airway resistance and total airway compliance measurements for similar experimental groups between experiment 1 and experiment 2, at both baseline and at increasing doses of methacholine (MCh)

Experiment		Baseline values	0 mg/mL	12.5 mg/mL	25 mg/mL	50 mg/mL	100 mg/mL
Total airway resistance							
1	Inf B-PBS	0.5512 ± 0.0345	0.5795 ± 0.0371	1.0437 ± 0.0672	1.6699 ± 0.1148	2.0632 ± 0.1552	2.2268 ± 0.1340
2	Inf B-PBS	0.5881 ± 0.0174	0.6421 ± 0.0187	1.3302 ± 0.0992	2.0525 ± 0.2234	2.2647 ± 0.2107	3.2423 ± 0.2404
1	Inf B-HDM	0.5989 ± 0.0244	0.6544 ± 0.0324	1.2302 ± 0.1445	2.5534 ± 0.4232	3.1823 ± 0.3079	6.0280 ± 1.1228
2	Inf B-HDM	0.5711 ± 0.0488	0.6334 ± 0.0519	1.3573 ± 0.1804	2.6771 ± 0.4078	3.1805 ± 0.5177	4.7392 ± 0.8228
1	Inf A-PBS	0.5523 ± 0.0124	0.6127 ± 0.0221	1.0951 ± 0.0490	1.8097 ± 0.1454	1.8817 ± 0.1277	3.1757 ± 0.5439
2	Inf A-PBS	0.5647 ± 0.0192	0.6226 ± 0.0172	1.1888 ± 0.1312	1.8694 ± 0.1336	1.8123 ± 0.0531	2.8665 ± 0.2331
1	Inf A-HDM	0.5361 ± 0.0220	0.6037 ± 0.0361	1.0237 ± 0.1123	2.2247 ± 0.5509	2.5345 ± 0.3490	2.9607 ± 0.3431
2	Inf A-HDM	0.5393 ± 0.0181	0.6040 ± 0.0202	1.0292 ± 0.0896	2.0994 ± 0.2909	3.1587 ± 0.3845	3.6473 ± 0.4978
Total airway compliance							
1	Inf B-PBS	0.0335 ± 0.0015	0.0324 ± 0.0013	0.0277 ± 0.0008	0.0236 ± 0.0014	0.0207 ± 0.0009	0.0172 ± 0.0012
2	Inf B-PBS	0.0345 ± 0.0018	0.0329 ± 0.0018	0.0253 ± 0.0014	0.0225 ± 0.0018	0.0203 ± 0.0017	0.0150 ± 0.0009
1	Inf B-HDM	0.0335 ± 0.0020	0.0315 ± 0.0019	0.0260 ± 0.0013	0.0184 ± 0.0013	0.0161 ± 0.0014	0.0105 ± 0.0016
2	Inf B-HDM	0.0358 ± 0.0026	0.0335 ± 0.0025	0.0275 ± 0.0023	0.0200 ± 0.0025	0.0154 ± 0.0019	0.0121 ± 0.0017
1	Inf A-PBS	0.0350 ± 0.0013	0.0332 ± 0.0013	0.0275 ± 0.0012	0.0232 ± 0.0014	0.0213 ± 0.0018	0.0166 ± 0.0019
2	Inf A-PBS	0.0341 ± 0.0017	0.0321 ± 0.0013	0.0264 ± 0.0016	0.0229 ± 0.0008	0.0223 ± 0.0011	0.0166 ± 0.0018
1	Inf A-HDM	0.0349 ± 0.0019	0.0324 ± 0.0021	0.0273 ± 0.0026	0.0211 ± 0.0028	0.0179 ± 0.0021	0.0139 ± 0.0018
2	Inf A-HDM	0.0371 ± 0.0017	0.0349 ± 0.0016	0.0292 ± 0.0014	0.0206 ± 0.0021	0.0147 ± 0.0014	0.0124 ± 0.0012

Lung inflammation was again evaluated by total cell counts and differential counts in BALF and by lung histopathology. No differences were found in the amount or type of cell infiltration in the BAL when comparing Infant B to Infant A mice given the same treatment (PBS or HDM) (Fig. 8I). Again, the amount of cell infiltration increased significantly after HDM treatment for mice with both microbiotas with a predominance of eosinophils over lymphocytes and neutrophils (Fig. 8I). Total cell numbers were also similar to what was observed for Infant A and Infant B in experiment 1 (Fig. 8B). Lung histopathology scores in experiment 2 showed significant inflammation in mice given HDM compared to PBS for Infant A and B microbiotas (*P* < 0.05) (Fig. 8D). No significant differences were observed in Infant A versus B when comparing the two PBS groups or the two HDM groups. The amount of mucus cell metaplasia in all mice given HDM was increased compared to mice given PBS, but no differences between microbiotas were found (Fig. 8E). Similarly, significant increases were observed in mucus cell metaplasia in HDM- compared to PBS-treated groups (*P* < 0.05) (Fig. 8E). Finally, significant differences in the density of eosinophils in HDM- versus PBS-treated groups were seen (*P* < 0.05), but not when comparing mice in like groups with different microbiotas (Fig. 8F). Overall, results indicate increased lung inflammation after HDM treatment, with no differences between the two microbiota groups. Small differences in findings between experiment 1 and 2 are explained by increased intragroup variances in experiment 1 and the larger number of experimental groups affecting statistical analyses. Thus, we can conclude that the values of baseline IgE for mice with the same microbiota were similar between the two experiments. The average value of baseline IgE for the Infant A PBS-treated group was 39 ng/mL, and for the Infant B PBS group, it was 242 ng/mL, which represents similar values when compared to experiment 1 (Fig. 9D and E). This shows that mice carrying Infant A or Infant B microbiota maintain their specific IgE phenotype throughout subsequent generations because microbiotas are passed on unchanged.

## DISCUSSION

Two new murine models with ^HU^microbiota from infants of the IOW birth cohort were established to study effects of gut microbiota on asthma pathogenesis. Fecal microbiotas from Infant A eczemic infants and Infant B non-eczemic infants were successfully transplanted to germ-free mice, remained stable, passed to offspring largely unchanged, and maintained a consistent diversity. To evaluate inflammatory allergic responses in airways of mice carrying different microbiotas, we used an induced asthma-like model using HDM, a common allergen associated with human asthma known to generate a Type 2 immune response polarization. This response is characterized by increased T helper 2 cytokines IL-4, IL-5, and IL-13, induction of class switch recombination in activated B cells to IgE; and eosinophilic infiltration into the lungs (43). Despite having distinct microbiotas when compared to ^MO^microbiota controls, Infant A, Infant B, and Adult C models all had decreased lung function at baseline without any allergen treatment when stimulated with methacholine in a dose–response design. Significant increases in Rrs and Rn at baseline were seen in mice with Infant A and Adult C microbiotas. Significant decreases in compliance (Crs) at baseline were seen in all mice with ^HU^microbiotas, which correlated with modest increases in cellular infiltrates in lungs. These results indicated a direct effect of ^HU^microbiotas eliciting increased Rrs, elastance (Ers), central airway resistance (Rn), tissue damping (G), tissue elastance (H), and decreased lung compliance. When mice were sensitized with HDM, significant differences were seen in lung baseline mechanics in Ers, G, H, and decreased compliance (Crs), but ^HU^microbiotas had less effect on Rrs and Rn in mice. Nevertheless, when sensitized with HDM, mice carrying all ^HU^microbiotas demonstrated AHR compared to PBS controls with ^MO^microbiota. Overall, similar results from experiments 1 and 2 support rejection of our hypothesis that Infant B microbiota would decrease allergic responses when compared to Infant A microbiota, the negative control (Mouse microbiota), and the positive control (Adult C microbiota). All of these ^HU^microbiota models will be useful for studying how acquisition and maturation of the microbiota affect an individual’s respiratory mechanics and immune responses. A great advantage to this comparative approach was using germ-free mice of a single C57BL/6 genotype so that host genetic background was held constant. Also, models were established without using antibiotics to deplete the original microbiota, which eliminates previous microbial influences on immune responses. Furthermore, the offspring we used as experimental mice acquired microbiota vertically and passively from their parents and littermates, similar to how a child would acquire its microbiota from parents and siblings. Because these microbiotas were vertically transferred largely unchanged to their offspring, they provide replenishable and tractable models for testing effects of microbiota on allergic airway disease outcomes. Strict exclusion of extraneous microbiota was achieved because mice were maintained in a closed barrier colony with sterile gowning and gloving for handling the mice. Because most work done on the influence of gut microbiota points to an early life effect, using a model where the microbiota establishes and matures naturally before mice are tested for asthmatic responses is an advantage for mimicking the human life course that predisposes to asthma.

### In mice with all three ^HU^microbiotas, we found significant impairment in baseline respiratory mechanics—in the absence of a direct stimulus for bronchoconstriction—when compared to the control ^MO^microbiota and to healthy nonallergic animals

To our knowledge, this is the first demonstration of impairment of baseline lung function attributed to specific human gut microbiotas. The differences in lung function at baseline between mice carrying ^HU^microbiotas versus congenic mice carrying ^MO^microbiota point toward a direct effect of gut microbiota on respiratory mechanics. This direct effect may be mediated by early microbiota during immune system education, which subsequently contributes to susceptibility to AHR. This conclusion is strengthened by findings that these three ^HU^microbiota models did not show the same magnitude of change in lung function parameters, tissue elastance, or central airway resistance. In a recent study by Brown et al. examining lung responses to ozone in C57BL/6 mice from two different vendors with different gut microbiotas, we noticed differences in pulmonary baseline responses even in the absence of ozone (44). Although the authors do not comment on these data, it provides another example where changes in baseline respiratory mechanics provoke differences in AHR between mice with different gut microbiotas, reinforcing the idea that gut microbiota has a direct effect on lung function. Because differences observed at baseline respiratory mechanics within each lung function may mislead one to believe that differences observed at different MCh doses are caused by increased reactivity to MCh or AHR, and not just due to the initial differences found at baseline, we developed a statistical model to evaluate reactivity to MCh while controlling for baseline in order to accurately pinpoint differences in AHR of these mice. Thus, we were able to account for differences at baseline respiratory mechanics when measuring AHR in mice exposed to PBS or HDM. The significant effects seen here on baseline respiratory mechanics support the idea that investigators should define and control for gut microbiota in studies where lung function parameters are evaluated.

### Taken together, BALF cell counts, histopathological scoring, cell-specific staining, and cytokine analyses suggest that inflammation, while elevated, is involved but is not sufficient to account for decreased lung function in mice with ^HU^microbiotas

This conclusion is further supported by the presence of lung function decline in all ^HU^microbiota mice in the absence of HDM sensitization (nonallergic), or in HDM-treated mice only 2 weeks after sensitization at a time when a full adaptive immune response has not occurred. Interestingly, ^HU^microbiota effects on the lungs were site specific and repeatable across experiments. For example, mice carrying Infant B microbiota had the highest AHR characterized by increased resistance of smaller peripheral airways (G) as well as an increased in airway closure (H), while resistance of the large conducting airways (Rn) likely played a lesser role. These studies confirm what has been seen in some patients with asthma, where AHR can be present in the absence of significant inflammation (45).

### Data support multiple allergy agonists and pro-inflammatory taxa functioning in a community-wide fashion to impair lung function in the absence of antagonistic and anti-inflammatory taxa

The summary heatmap (Fig. 10) shows that this concept is strongly supported and that each ^HU^microbiota harbored a different constellation of agonists and pro-inflammatory taxa, which likely lead to the varied allergic and pathologic manifestations measured. Thus, the pattern of responses in each microbiota correlated to the presence of different mixes of agonist and inflammatory taxa. For example, Infant A mice, where lung function responses were less than in mice with Infant B and Adult C microbiotas, had much smaller proportions of allergy agonists *Alistipes*, *Sutterella*, and *Parasutterella,* and a larger proportion of the allergy antagonist *Bifidobacterium*. Also, Infant A microbiotas had much smaller proportions of pro-inflammatory taxa *Parabacteroides* and *Parasutterella,* and a larger proportion of anti-inflammatory *Bifidobacterium*. Finally, diversity had no apparent effects as the Infant A microbiota had low diversity, and these mice had the lowest positive allergic responses when compared to Infant B and Adult C.

We acknowledge that there could be a single bacterial taxon that exerted large effects because several potential respiratory pathogens were identified, but none that solely explain the data. Thus, our results lead us to reject our hypothesis that *E. coli-Shigella* was the sole responsible agonist taxon, given that this taxon was present in appreciable amounts only in the Infant A microbiota. Another possibility is that metabolites from groups of taxa may act to impair lung function in mice with the ^HU^microbiotas. Because of the complexity of the mixes of allergy agonists/antagonists and pro- and anti-inflammatory taxa in the three ^HU^microbiotas that were associated with lung function decline, work is underway that shows these microbiotas are metabolically distinct. Yet, more work is needed to discern whether the metabolites produced by these three human microbiotas are mainly responsible for these effects or whether specific virulence mechanisms drive the phenomena. Other studies have shown the association of certain gut microbiota compositions, diversity, or metabolites with the presence of allergic airway disease (46, 47). While mechanisms are not yet known, Olszak et al. showed that microbial exposure during early life has persistent effects on natural killer T cell function (46). Depner et al. mentioned the association of increased levels of butyrate produced by the microbiota as a potential clue to how specific microbial communities exert an asthma-protective effect (32). Because butyrate and other metabolic products can be sensed by the vagus nerve as described in the process of inflammatory bowel disease (48), it is likely that either the presence or the absence of certain gut metabolites may have effects on the afferent and efferent nerve output, which innervate the airways and chest wall and travel via the vagus nerve. These nerve terminals are responsible for the degree of airway smooth muscle constriction and AHR, especially in response to increasing doses of MCh, and they exhibit plasticity in early stages of life when certain conditions are met, such as an increase in the amount of specific neurotrophins and nerve growth factors.

### Known allergy antagonists and anti-inflammatory bacterial taxa dominated ^MO^microbiota while the presence/absence and abundances of these genera were minimal in ^HU^microbiotas

Allergy antagonists unique to the ^MO^microbiota included *Clostridia (Clostridiales), Faecalibacterium,* and *Ruminococcus-Coprococcus,* while *Roseburia, Lactobacillus, and Bifidobacteria* were in high amounts but also present in one of the human-derived communities. Anti-inflammatory bacterial taxa were also found exclusively in the protected ^MO^microbiota including *Roseburia* and *Lactobacillus,* which were distinct OTUs from the same genera seen in Infant B microbiotas. These observations are consistent with the findings of Arrieta and colleagues in children at risk of asthma, which were also missing some of these protective taxa (*Lachnospira*, *Veillonella*, *Faecalibacterium*, *Rothia*) (22). Yet, genera such as *Veillonella* contain both protective and pathogenic species, so more work is needed to determine species-specific characteristics of the identified bacterial taxa. Depner et al. showed that microbial communities with the likelihood of producing butyrate—especially those in children on farms—were the ones associated with the highest protective effect against asthma (32). They demonstrated that bacterial taxa contributing to protective effects of butyrate included *Roseburia* and *Coprococcus* (32). In our study, *Roseburia* and *Coprococcus* were present in protected mice with ^MO^microbiota but were also found in similar levels in Infant B and Adult C mice bearing ^HU^microbiotas. Moreover, mice with Infant B microbiota hypothesized to be “protective” had decreased lung function and the highest AHR compared to the control ^MO^microbiota. This may have been due to the shifts in abundance observed in bacterial taxa in the transplanted mice compared to donor infants; all mice were fed a high-fiber, low-fat diet mouse chow substantially different from the milk and/or formula diet of donor infants. We did examine the asthma status of infants who donated fecal samples for transplant; all were as expected except for one “No eczema” infant that had a single status recording of asthma at 6 years of age, which may or may not have affected their fecal donation at 3 months of age. At this point, this child has not experienced additional episodes. Metabolomic analysis is underway on these infants and mice that shows differences between those with AHR and the negative controls. Trompette et al. treated mice with the short-chain fatty acid (SCFA) propionate and found alterations in bone marrow hematopoiesis with enhanced macrophage and dendritic cell (DC) precursors and subsequent seeding of the lungs by DCs with high phagocytic capacity but impaired ability to promote Type 2 effector functions (49). However, our early metabolomic findings suggest that it may be more complicated than simply a difference in SCFAs. Regardless, we rejected our hypothesis that a high-abundance *Bacteroides*-enriched gut microbiota from non-eczemic infants was protective in transplanted mice after challenge with HDM. More work is needed to determine if adding back each or all of these organisms in early life could prevent decreases in lung function in mice with ^HU^microbiotas.

Thus, lung function declines in mice with what we expected to be a protective Infant B microbiota were clear-cut, but the cause for this outcome was not. We acknowledge it was more difficult to discern impairments in lung function due to a specific microbiota when examining mice sensitized with HDM, because increased allergen-associated inflammatory responses were high across all microbiota groups. After HDM treatment, inflammation around the airways caused an overall increase in resistance and tissue stiffness, making subtle differences in respiratory mechanics observed at baseline in the negative control mice harder to detect. However, it is known that HDM has no effect on baseline respiratory mechanics as seen in studies of HDM-treated BALB/c mice (50). Therefore, this study suggests the AHR differences found between mice carrying the ^HU^microbiotas and the control mice are due to a direct effect of the microbiota on airway structural components and potentially airway innervation. Yet, we cannot rule out action by other immune cells known to influence airway innervation and AHR that were not evaluated, such as mast cells (51–54). Mast cells may provide an intermediary pathway between gut microbiota and lung function responses (54, 55).

## MATERIALS AND METHODS

### Laboratory animals

We followed the National Institutes of Health Guide to the Care and Use of Laboratory Animals. All protocols were reviewed and approved by the Institutional Animal Use and Care Committee of Michigan State University (06/12-107099, 6/15-101-00, and 05/17-091-00). We used C57BL/6 mice originally obtained from The Jackson Laboratory (Bar Harbor, ME, USA) barrier facility in one purchase. Mice used had conventional ^MO^microbiota or ^HU^microbiota from (i) 3-month-old infants of the IOW birth cohort (12) or (ii) young adults (Adult C) (39, 40). Mice with human microbiota were offspring of mice given FMT from IOW infants. All experiments were conducted with age-matched male and female mice between 8 and 10 weeks of age. Specific-pathogen-free breeding colonies were established at Michigan State University in facilities free of *Helicobacter*, *Campylobacter*, *Citrobacter rodentium*, *Enterococcus faecalis*, and other known colitis-causing or respiratory pathogens based on testing (39). Sentinel mice were used in ^MO^microbiota and ^HU^microbiota mouse colonies to monitor for known mouse pathogens at 6-month intervals (IDEXX BioAnalytics, Columbia, MO, USA). All mice were managed under biosafety level 2 regulations.

### Study cohort and collection of fecal samples from infants

Fecal samples from 3-month-old infants of the IOW third generation birth cohort were used (12). Sixty infants were recruited by obtaining informed consent from pregnant IOW cohort members and pregnant partners of male cohort members to collect fecal samples and clinical outcome parameters at 3, 6, 12, 24, and 36 months of age. Ethics approval was obtained from the local/national ethics committees at recruitment of the birth cohort between January 1989 and February 1990 and subsequently at each assessment. Children were recruited into the “Third Generation Study” under ethics approval numbers 09/H0504/129 (22 December 2019), 14/SC/0133 (22 December 2019), and 14/SC/1191 (15 November 2016) from the University of Southampton, Southampton, UK. Data analysis was carried out without the knowledge of the personal identity of the infants. Fecal samples were collected from diapers by nurses, coded to protect identity and clinical status, and placed into a sterile container at the 3-month visit. If a fecal sample was not available during the clinic visit, nurses arranged a home visit to collect samples. Samples were stored on ice for the short transport, bagged in an anaerobic pouch, and stored at −80°C until processed. Infant fecal samples for transplant into germ-free mice were selected based on 16S rRNA gene sequencing and correlation with assessments for allergic disease including eczema, wheeze, atopy, and asthma over the 3-year time frame. Samples used for FMT were collected previouslyand selected based on (i) bacterial taxonomic structure at genus level, (ii) ordination using CCA, and (iii) SIMPER analysis. All human samples were handled under biosafety level 2 regulations.

### Fecal collection, 16S rRNA gene sequencing, and data analysis to select samples for FMT

Samples were transported on dry ice and handled in an anaerobic hood for aliquoting. DNA was isolated from 500 mg of each sample using the FastDNA SPIN Kit for stool (MP Biomedicals, Solon, OH, USA) according to the manufacturer’s protocol. Careful attention was given to preventing lab contamination by the operator or within the laminar flow hood. All reagents were validated free of 16S DNA before use. Standard controls (mock community, ATCC/BEI) were run alongside samples to control for contamination of reagents. Qubit analysis (Thermo Fisher Scientific, Waltham, MA, USA) was performed to determine DNA concentration; 16S rRNA gene PCR was performed to validate the quantity and purity of DNA samples prior to submission to the Michigan State University Research Technology Support Facility. The V4 region of the 16S rRNA gene was amplified by PCR in triplicate using two sets of barcoded primers (56); PCR products were purified, combined, and sequenced using Illumina MiSeq. Sixty-two samples were submitted for sequencing, 60 mouse samples, the original fecal slurry used for inoculation of founder mice, and a mock community (HM-782D, BEI) for estimation of sequencing error. PCR products were normalized using an Invitrogen SequalPrep DNA normalization plate and the normalized products pooled. After quality control and quantitation, the pool was loaded on a standard MiSeq v2 flow cell and was sequenced with a 500 cycle MiSeq v2 reagent kit (paired-end 250 base pair reads). Base calling was performed by Illumina Real Time Analysis (RTA) v1.18.54, and output of RTA was de-multiplexed and converted to FastQ format files with Illumina Bcl2fastq v1.8.4. High-quality reads (109,085) from Illumina sequencing remained—average read number per group: 3,896 (min 3,245, max 5,053).

16S rRNA gene amplicon analysis protocol was performed using QIIME2 (v. 2019.1) version, https://docs.qiime2.org/2019.1/, accessed 5/01/2019. Alignment was carried out using the Silva 16S ribosomal gene database (57). Chimeric sequences, chloroplast, mitochondria, Archaea, or Eukaryota sequences were removed using UCHIME. Sequences were clustered in OTUs of 97% sequence identity yielding 128 OTUs. Analyses were performed in PAST 3.07 (58) and R version 4.06 (59). After processing sequences and chimera removal in QIIME2, groups were sub-sampled to a 3,245 read depth and grouped by an average neighbor method.

### Preparation of the inocula

To preserve all components of the microbiota in its original state, fecal samples and the inocula were handled in an anaerobic hood. Each sample was suspended in 2 mL of Trypticase soy broth. Samples for each microbiota mixture were combined in a single 50 mL conical centrifuge tube, solids were removed by sedimentation, and the supernatant was removed to a sterile tube. Inocula-containing tubes were sealed prior to removal from the anaerobic hood and were transported on ice to the containment facility. The inoculum (200 µL) was delivered to each mouse immediately by oral gavage in a laminar flow biological safety cabinet using a 3.5 French red rubber feeding tube attached to a 1 mL syringe. Approximately 0.5 mL of each Infant A and Infant B mix was saved at −80°C for sequencing of the inocula.

### FMT of germ-free mice

C57BL/6 germ-free mice from the barrier facility at The Jackson Laboratory underwent caesarian rederivation, and their offspring were thereafter propagated in the germ-free facility at the University of Michigan (Ann Arbor, MI, USA). C57BL/6 mice with conventional ^MO^microbiota were obtained from the same facility at the same time and maintained to prevent acquisition of extraneous microbiota. Germ-free C57BL/6 mice for these studies were bred by the University of Michigan. At 7 weeks of age, mice were transported to the Michigan State University in sterile shipping containers, immediately placed into a biosafety cabinet, inoculated with the human FMT by oral gavage, and placed into sterile cages with sterile food, water, and filtered air. All operators including scientists and animal care personnel gowned and gloved using sterile technique every time mice were handled to prevent transfer of operators’ or environmental microbiota to mice. Five male and five female germ-free C57BL/6 mice received a fecal transplant of each microbiota, Infant A or Infant B inoculum.

Mice were observed for clinical signs twice daily for 2 weeks after FMT. Then, they were placed in breeding pairs according to their microbiota and were allowed to breed for at least two breeding cycles to generate mice for a closed colony and to conduct the two experimental studies. Breeding practices were employed to maximize mixing of the microbiota within a microbiota type: (i) male mice were left with the pregnant female until the offspring were weaned, and (ii) we randomized selection of males for breeding pairs to eliminate a “family” bias in subsequent generations. Adult C mice were transplanted in a similar manner as previously described (39, 40). All mice were monitored by a rigorous testing program for mouse pathogens using sentinels and testing by Charles River Laboratories (Wilmington, MA, USA).

### Experimental designs for mouse model of allergic airway disease

#### Experiment 1 design

To address the effect of microbiota on airway allergic responses, 20 mice, from each of the four different gut microbiota groups including mouse (control), Infant A, Infant B, and Adult C, were randomly assigned to treatment groups with vehicle (PBS) or HDM as previously described (60). Outcomes assessed in each of these experiments (airway responsiveness, histopathology scores, BALF cytology, serum immunoglobulins, and cytokines) are quantitative measures of allergy that were analyzed as continuous variables.

Age-matched C57BL/6 mice (*n* = 10 per group) were used in all experiments. We used inbred mice housed in a strictly controlled environment and handled in a highly standardized manner, which improved uniformity of allergy response variables within groups. This uniformity of outcome variables has long been our experience, is widely established in the literature (1, 3–7), and was the basis for our sample size. The sample size was selected based on achieving 83.1% power for AHR to MCh after HDM treatment. A total of eight experimental groups (*n* = 10 per group, 5 males and 5 females) were used. At the end of the HDM treatment protocol, data were collected from each mouse to evaluate allergic responses based on lung function and immune responses. Mice that were mis-dosed during the treatment protocol (four mice in Infant B PBS group) and mice that died during the surgical procedure or during measurement of lung mechanics for lung function were excluded from the analysis.

#### Experiment 2 design

To confirm findings that Infant A and Infant B microbiotas both acted to decrease lung function experiment 1, we conducted a second experiment. The design was exactly the same as experiment 1, except we used only mice carrying Infant B or Infant A microbiota. Mice were sensitized to HDM as indicated in experiment 1. Lung function and MCh challenge, lung inflammation, lung histopathology, and differential BALF cell counts were performed as described in experiment 1. For experiment 2, lung histopathology scoring was evaluated in three categories: inflammation, mucus cell metaplasia, and density of eosinophils. We measured mucus cell metaplasia in this experiment as another possible inflammatory marker for the degree of the airway allergic response, with the intention of picking up other differences between responses in mice with Infant A and Infant B microbiota.

### Sensitization to allergen

Approximately 10–15 weeks after birth, house dust mite allergen sensitization was initiated (Table 3). Mice were inoculated on a schedule such that those receiving allergen were exposed to 50 µg of HDM in 25 µL of Dulbecco’s PBS on the first day, designated day 0, and 30 µg of HDM in 30 µL of PBS on days 2, 5, 7, 9, and 12 (Table 3). Those controls receiving only PBS received equivalent volumes on those days. To perform the sensitization, mice were placed in an induction chamber and given 3% isoflurane through a vaporizer at a rate of 1 L/min until non-motile and non-responsive to handling. Mice were then removed from the chamber, held by scruffing, and had 10 µL of the HDM solution or PBS administered intranasally via a pipette. Between each subject, the isolation chamber and all gloves were sprayed with 70% ethanol.

### Tracheal cannulation and respiratory mechanics evaluation using flexiVent analysis

Forty-eight hours after receiving the last dose of HDM or PBS, mice were anesthetized via a mixture of ketamine (range 80–120 mg/kg), xylazine (range 8–12 mg/kg), and acepromazine (range 5–10 mg/kg) given intraperitoneally. Once movement ceased and mice were non-responsive to a toe pinch, a ventral midline incision was made over the mid-cervical region from the base of the jaw to the thoracic inlet. The trachea was exposed by blunt dissection, a small incision was made in the mid-cervical trachea distal to the larynx, and an 18-gauge beveled cannula was inserted and secured with a ligature. Animals were then ventilated using a flexiVent apparatus (SCIREQ, Montreal, QC, Canada) at a frequency of 150 breaths per minute and at a volume of 10 mL/kg of body weight at 0.17–0.2 mL/breath. Mice were allowed to exhale passively through an expiratory valve to an end pressure of 4 cm H_2_O positive end expiratory pressure. Any animals that displayed respiratory effort during the process were administered an additional 0.1 mL injection of ketamine (range 80–120 mg/kg), xylazine (range 8–12 mg/kg), and acepromazine (range 5–10 mg/kg) given intraperitoneally. To stimulate airway constriction, an allergic challenge of aerosol acetyl-β-methylcholine (MCh, Sigma-Aldrich, St. Louis, MO, USA) was generated through an in-line nebulizer (Aeroneb, SCIREQ, Montreal, QC, Canada) that was administered in increasing doses (0.625 to 200 mg/mL) in 10 seconds of dosing intervals with a 3- to 5-minute break between dosing. Six increasing doses of MCh starting at 0 (PBS), 6.25, 12.5, 25, 50, and 100 mg/mL were delivered. Data were collected for 2 minutes 45 seconds following each methacholine dose in a series of 12 repetitions of the single frequency forced oscillation (SnapShot-150) and complex forced oscillation (Quick Prime) maneuvers as applied by the flexiVent apparatus. Data on pressure and flow were collected throughout and used to determine resistance and compliance of the airways. Responses to increasing methacholine doses were measured within 2 minutes 45 seconds post-nebulization using the flexiVent. Single frequency or complex forced oscillations maneuvers were applied by the flexiVent. Volumes and pressures were recorded, and the parameters measured or derived from those measurements to evaluate lung function include total resistance of the respiratory system (Rrs) (resistance to air flow as a function of the extent of airway constriction); dynamic compliance of the respiratory system (Crs) (the ability of the lungs to stretch); dynamic elastance (Ers) (lung stiffness); Newtonian resistance (Rn) (central airway resistance constriction); tissue damping (G) (tissue [alveolar] resistance); and alveolar tissue constriction and tissue elastance (H) (tissue [alveolar] elastance [alveolar tissue stiffness]).

### Bronchoalveolar lavage (BAL) total cell and differential cell counts

To collect BALF, 0.7 to 0.8 mL of sterile saline was slowly instilled through the tracheal catheter. Then, BALF was slowly withdrawn, dispensed into a sterile tube, and the process was repeated. BALF samples were pooled, and total cells were counted on a hemocytometer. Thereafter, 150 µL BALF was centrifuged onto slides using a Shandon Cytospin 3 Centrifuge (Marshall Scientific, Hampton, NH, USA) at 40 × *g* for 10 minutes. Slides were air dried, fixed in methanol, and stained with Diff Quick stain (Polysciences, Inc., Warrington, PA, USA) for differential counting. The remaining BALF was centrifuged at 465 × *g* (=1,500 rpm), at 4°C for 15 minutes, and supernatants were removed and stored at −80°C.

### Euthanasia

Mice still under anesthesia from the mechanics evaluation were euthanized via exsanguination. Mice were wet with 70% ethanol to avoid hair in the incision site, and then a ventral midline incision was made from the xiphoid process to the pubis. Visceral organs were evaluated for gross abnormalities then moved to the side to expose the abdominal aorta. A 1 mL syringe with a 25gG needle was used to collect approximately 0.5 mL of blood, after which the vessel was severed to complete exsanguination. Collected blood was transferred to a 0.8 mL lithium heparin separator tube, centrifuged at 3,500 rpm at 4°C for 15 minutes, and serum was removed and stored at −20°C.

### Lung dissection, fixation, sectioning, and staining

Once BALF was collected, the thoracic cavity was opened by blunt dissection of the hemi-diaphragms, and complete collapse of lungs was checked before removal of the chest wall. Thereafter, the heart, lung, and trachea were removed enbloc with extreme care to prevent nicking of trachea, extrapulmonary bronchi, or lung lobes. Lungs were removed from the thoracic cavity attached to the canula and placed on a sheet of dental wax to reduce surface friction. First, the right main stem bronchus was ligated using silk-suture, and the four right lung lobes were removed. The right cranial, middle, and postcaval lobes were placed into a 2 mL screw-top cryovial, snap frozen in liquid N_2_, and stored at −20°C. The right caudal lobe was cut in half, placed into a 2 mL flat-bottom tube in 1.5 mL of RNAlater, and refrigerated at 4°C refrigerator for 24 hours followed by storage at −20°C. The trachea and left lung lobes were fixed in 10% neutral buffered formalin using an inflation-fixation apparatus. Trachea and lungs were perfused under constant pressure (30 cm of fixative pressure) for 1 hour under 30 cm of fixative pressure, then they were tied off, removed, and stored in a large volume of formalin until further processing.

The fixed left lobes were separated from the trachea by severing the left main stem bronchus using scissors. The lobes were then sectioned on dental wax and sliced transversely into approximately 3 mM sections using a straight razorblade. These sections were placed cut side down into a cassette, which was then submerged into 30% ethanol. The specimens were dehydrated, embedded in paraffin, and three sections were cut from each block at 4 µm thickness. Sections were then deparaffinized and stained with hematoxylin and eosin (H&E), Alcian blue periodic acid-Schiff (ABPAS), or major basic protein (MBP) by the Investigative Histopathology Laboratory, Division of Human Pathology, Department of Physiology, Michigan State University. Slides were then scanned and digitized using an Olympus VS110 machine and were subsectioned at 20 × using Visiomorph Microimager (Visiopharm, Denmark) (61). In order to score inflammation, slides stained for hematoxylin and eosin were assessed. Six 10× fields under were captured using a Nikon DS-F13 camera attached to a Nikon H600L light microscope and stitched together using the NIS-Elements BR 5.02.00 program (Nikon-USA, Melville, NY, USA). Within these fields, large airways, small airways, and vasculature were assessed for the layers of inflammatory cells that surrounded each structure at their densest/thickest point. The results for each type of structure were averaged, and then the three resulting values were averaged for an overall inflammatory score.

### Lung histopathology scoring

The histologic scoring system for the lungs was based on published scoring criteria (62–66). Our scoring system was based on a meta-analysis of four published protocols allowing for focus on observed lesions. We first noted the number of tissue sections on the slide. Using a battlement pattern for each section, the total number of large airways was recorded. The reader then repeated the battlement pattern evaluating how many of these airways were affected by inflammation. To be considered positive, an airway was required to be cuffed with closely associated mononuclear cells. Thereafter, the large airway(s) with the worst pathology was evaluated to determine the density/thickness of inflammation surrounding the structures. This evaluation was based on the thickness of the layer of inflammatory cells comprising the diameter of the aggregate that was recorded as a ranked score (0–3, none, mild, severe) based on the rubric for those structures. We also noted the diameter of the largest aggregate evaluated. The process was repeated (steps 1–4) for small airways and for vasculature (steps 1–4). Thereafter, the parenchyma was evaluated for the presence of inflammatory cells not associated with airways or vasculature, and a score was assigned (0–3, none, mild, severe) for generalized inflammation. Next, a point of 1 was added if focal clusters of inflammatory cells were present within the parenchyma. Finally, a point of 1 was added if there was evidence of obliterated small airways within areas of dense parenchymal inflammation (either focal or generalized). A total score was reached by adding all the points scored to achieve a “pathology grade.” Two independent blinded investigators (L. S. Mansfield and I. A. M. Uribe) scored the images based on the consensus scoring system and graded the inflammation score.

### Measurement of lung cytokines

The cranial right lung lobes were thawed on ice, weighed, and homogenized in Q-mammalian protein kit buffer containing protease inhibitor (QIAGEN Inc., USA), using a TissueLyser II (QIAGEN Inc., Germantown, MD, USA). Homogenates were centrifuged at 14,000 × *g* for 20 minutes in a microcentrifuge precooled to 4°C, and supernatants (i.e., total protein fractions) were aliquoted into new microcentrifuge tubes precooled to 4°C and stored at −80°C. Total protein in mg/mL was measured using the Pierce BCA Protein Assay Kit (Thermo Fisher Scientific Inc., Waltham, MA, USA). Cytokines were measured using a commercially available flow cytometry-based, multiplexed, bead assay panel (LEGENDplex Mouse Th Cytokine Panel, BioLegend, San Diego, CA, USA). Just before analysis, aliquoted supernatants of the lung homogenates were thawed on ice and centrifuged at 300 × *g* for 10 minutes at 4°C to further pellet any debris and ensure clarity. The assay was performed using undiluted supernatant and a V-bottom microplate, according to the manufacturer’s instructions. All samples were run in duplicate. Analytes included IFN-γ, TNF-α, IL-2, IL-4, IL-5, IL-6, IL-9, IL-10, IL-13, IL-17A, IL-17F, IL-21, and IL-22. Data were acquired on a BD FACSCanto II flow cytometer and were analyzed using the LEGENDplex Data Analysis software, where standard curves were generated for each analyte. Data are presented as the average of the replicates in picograms of cytokine per milligram of protein.

### Total IgE and specific anti-HDM IgE analysis

Plasma samples were aliquoted to avoid repetitive freezing and thawing, and were stored at −80°C. Total plasma IgE was measured using BioLegend Mouse IgE ELISA MAX Deluxe Set (BioLegend, Inc. San Diego, CA, USA). Briefly, samples were diluted according to a standardized dilution factor based on results from preliminary assays. Samples that were higher than the limit of detection on the first run were repeated using a higher dilution. Mouse IgE standard was reconstituted in buffer, and six twofold serial dilutions were performed according to the manufacturer’s instructions. All incubation steps with shaking were conducted at 200 rpm. Capture antibody for mouse IgE was diluted in 1× coating buffer. One hundred microliters was added to each well of a 96-well plate, and the plate was incubated overnight at 4°C. Plates were washed four times with ELISA wash buffer (20×) (BioLegend, Inc., San Diego, CA, USA), and 200 µL of 1× Assay Diluent A was added to each well for blocking. Plates were incubated for 1 hour at room temperature. Samples were diluted in 1× Assay Diluent A. Plates were washed four times, and 100 µL of each sample was added to appropriate wells. Plates were incubated at room temperature with shaking for 2 hours and then washed four times. Biotinylated detection antibody (100 µL) (diluted 1:200 in 1× Assay Diluent A) was added to each well, and plates were incubated for 1 hour at room temperature with shaking. Plates were washed four times. Avidin-HRP (100 µL) (diluted 1:1,000 in 1× Assay Diluent A) was added to each well, then plates were incubated for 30 minutes at room temperature with shaking. Plates were washed five times. Wash buffer was allowed to sit for 30–60 seconds between each wash. 3,3′,5.5′-Tetramethylbenzidine (TMB) substrate solution was prepared, and 100 µL of substrate solution was added to each well. Plates were then incubated in the dark at room temperature for 15 minutes. Stop solution (100 µL) (2 N sulfuric acid) was added to each well, and the absorbance was read at 450 nm using a microplate reader. All the samples were run in duplicate. Concentrations were calculated through elisaanalysis.com, using a four-parameter logistic curve as recommended in the kit instructions. For samples that were repeated on multiple plates, the concentrations were averaged. Data are presented as nanograms per milliliter.

For specific HDM-IgE measurement, we used the Mouse Anti-House Dust Mite (HDM) IgE Antibody Assay Kit (Chondrex Inc, Woodinville, WA, USA). Plasma samples were handled as above, and different dilutions were tested in preliminary assays to arrive at a standard dilution of 1:10. All samples were run in duplicate. The assay was performed following the manufacturer’s kit instructions. Final concentrations were calculated using a four-parameter logistic curve through elisaanalysis.com and are presented as the average of the duplicates in nanograms per milliliter.

### Bacterial DNA isolation from mouse feces

At the time of necropsy, fecal samples were collected, snap frozen on dry ice, stored at −80°C, and processed for 16S rRNA gene sequencing. DNA was extracted using the FastDNA SPIN Kit stool kit (MP Biomedicals, Solon, OH, USA). Briefly, for each mouse, a single fecal pellet was suspended in 978 µL of sodium phosphate, 122 µL of MT buffer was added, and the sample was homogenized using a bead beater for 40 seconds. The samples were centrifuged at 14,000 × *g* for 10 minutes, and the supernatant was then extracted, combined with 250 µL PBS, and centrifuged at 14,000 × *g* for 5 minutes. The supernatant was again collected and combined with 1 mL of the provided DNA-binding matrix. The resulting solution was mixed on a tube vortex for 2 minutes, allowed to resettle, and then 500 µL of supernatant was discarded. The remaining solution was resuspended and then centrifuged 600 µL at a time at 14,000 × *g* for 1 minute in a Spin Filter tube. The resulting pellet was washed by resuspending in 500 µL of ethanolated SEWS-M and centrifuged at 14,000 × *g* for 1 minute, then again for 2 minutes after disposing of the waste fluid. Samples were then allowed to air dry at room temperature for 5 minutes before being resuspended in 200 µL of DES (DNase/pyrogen-free water) and incubated at 55°C for 15 minutes on a heating block. Samples were then centrifuged at 14,000 × *g* for 2 minutes, and the filtered liquid was collected for further analysis. The presence of DNA was assessed in all samples using a Nanodrop instrument before being placed in a −80°C freezer for storage. DNA samples from all mouse fecal samples were validated and processed for 16S rRNA gene sequencing as described for infant fecal samples above. 16S rRNA gene amplicon analysis was performed using QIIME2 (v. 2019.1) as described above for infant fecal samples, and microbial ecology data were analyzed.

### Statistical analysis

Microbial ecology data from16S rRNA gene sequencing of infant and mouse fecal DNA were analyzed to determine the richness and evenness of the infant and mouse fecal bacterial communities. PCA was performed in R (59) to examine relative compositions of the three ^HU^microbiotas (Infant A, Infant B, and Adult C), ^MO^microbiota, and the two Infant inocula A and B. PCA was also performed to examine the relatedness of the three human-derived microbial communities Infant A, Infant B, and Adult C microbiotas separately. To examine similarity (dissimilarity) between bacterial taxa in these two sets of groups, we performed a two-way permutational multivariate analysis of variance (PERMANOVA) in PAST 4.03 using the Bray-Curtis similarity measure (with microbiota and treatment as factors). UPGMA was performed in PAST 4.03 on samples from all microbiota groups and the Infant A and Infant B inocula using the Bray-Curtis similarity measure with 1,000 bootstrap replications in PAST version 4.03. For the heat maps, we compared the average relative abundance of the bacterial taxa contributing 90% of the differences in Similarity Percentage calculation (SIMPER; in PAST 4.03) for (i) all samples (Infant A, Infant B, Adult C, ^MO^microbiotas, and the two Infant inocula A and B) and for the (ii) three ^HU^microbiotas.

For cytokine analysis, plasma total and specific HDM IgE, differential cell counts and lung histopathology, and lung function measurements are presented as the mean ± standard error. Kruskal-Wallis one-way analysis of variance (ANOVA) statistic was used on PAST 4.02 to compare baseline lung functions for the microbiota groups.

A general linear mixed (GLM) model was used to examine the effects of MCh concentrations, microbiota groups, and treatment assignment on the degree of AHR (67). Outcomes were measurements of the lung function at peak (bottom for Crs) after each MCh dose or after nebulized PBS was delivered (dose 0). The outcomes were included in the model with a logarithmic transformation. Predictors included dose, microbiota, treatment, and the log-transformed baseline values for each dose. Random effects accounted for the repeated measures on each mouse. ^MO^Microbiota group served as the reference category in microbiota groups, whereas PBS (sham) treatment served as the reference treatment. Interaction terms were included to assess the effect of dose on the peak lung function for each microbiota group. To compare reactivity to increasing doses of MCh, the predicted slopes coefficients from the linear mixed model for each microbiota group were compared using the Tukey test for multiple pairwise comparisons. For each model, we considered a *P* value < 0.05 to indicate statistical significance. All analyses were conducted using statistical software R version 4.06 using the glmmTMB and lme4 packages (59). Pearson and Spearman correlation analyses were conducted using the ggpairs and ggally packages in R software.

## Data Availability

All data presented within this article are available without restriction for use. Data sets are available through the DRYAD database under https://doi.org/10.5061/dryad.zkh1893m9.

## SUPPLEMENTAL MATERIAL

Supplemental Tables mbio.03764-24-s0001.docx Tables S1 to S5.

ASM does not own the copyrights to Supplemental Material that may be linked to, or accessed through, an article. The authors have granted ASM a non-exclusive, world-wide license to publish the Supplemental Material files. Please contact the corresponding author directly for reuse.
