# Supplementary material for: Fecal microbiota transplants (FMT) of three distinct human communities to germ-free mice exacerbated inflammation and decreased lung function in their offspring
Source: mBio. 2025 Apr 10;16(5):e03764-24. doi: 10.1128/mbio.03764-24 (PMC12077122; doi:10.1128/mbio.03764-24)
Supplement: Supplemental Tables — Tables S1 to S5. [file mbio.03764-24-s0001.docx]

**Supplemental Table 1.** Statistically significant differences in abundances of taxa associated with allergy or protection from allergy and/or gut inflammation or protection from inflammation in gut microbiotas of mice carrying different human fecal transplants (Figure 4B). Taxa are presented in alphabetical order because some have been associated with both allergic and inflammatory outcomes or protection from those outcomes and appear in more than one panel of Figure 4B. Kruskal Wallis nonparametric ANOVA with Benjamini-Hochberg adjustment for multiple comparisons followed by Mann Whitney pairwise comparisons with Bonferroni correction for multiple comparisons. NS, P>0.05; NA, pairwise comparisons not carried out because adjusted Kruskal Wallis P was >0.05.

|  |  | **Mann-Whitney pairwise P after Bonferroni correction** | | | | | |
| --- | --- | --- | --- | --- | --- | --- | --- |
| **Taxon** | **Kruskal Wallis P, Benjamini-Hochberg adjustment** | **Infant B vs Infant A** | **Infant B vs Adult** | **Infant B vs Mouse** | **Infant A vs Adult** | **Infant A vs Mouse** | **Adult vs Mouse** |
| *Actinobacteria_Bifidobacteriaceae_Bifidobacterium* | 1.15E-09 | 1.92E-05 | 0.04691 | 0.0004374 | 1.91E-07 | NS | 5.49E-06 |
| *Actinobacteria_Coriobacteriaceae_Collinsella* | 6.30E-10 | 2.10E-06 | 0.01402 | 2.10E-06 | 0.001002 | NS | 0.001002 |
| *Bacteroidetes_Porphyromonadaceae_Parabacteroides-a* | 4.10E-13 | 4.80E-08 | NS | 4.80E-08 | 1.80E-07 | NS | 1.80E-07 |
| *Bacteroidetes_Porphyromonadaceae_Parabacteroides-b* | 7.35E-14 | 2.09E-07 | 6.39E-07 | 9.09E-08 | 2.09E-07 | NS | 9.09E-08 |
| *Bacteroidetes_Prevotellaceae_Prevotella_9* | NS | NA | NA | NA | NA | NA | NA |
| *Bacteroidetes_Rikenellaceae_Alistipes-a* | 2.14E-12 | 3.44E-07 | 0.01284 | 3.22E-07 | 3.44E-07 | NS | 3.22E-07 |
| *Bacteroidetes_Rikenellaceae_Alistipes-b* | 8.39E-12 | 4.80E-08 | 0.03336 | 8.00E-08 | 1.80E-07 | NS | 2.78E-07 |
| *Firmicutes_Clostridiaceae_1_Clostridium_sensu_stricto_1-a* | 6.38E-04 | NS | NS | 0.001625 | NS | 0.001625 | 0.01817 |
| *Firmicutes_Clostridiaceae_1_Clostridium_sensu_stricto_1-b* | 2.21E-09 | 1.49E-07 | NS | NS | 4.80E-08 | 4.80E-08 | NS |
| *Firmicutes_Enterococcaceae_Enterococcus* | 2.84E-12 | 1.47E-07 | NS | NS | 1.47E-07 | 6.76E-08 | NS |
| *Firmicutes_Lachnospiraceae_Coprococcus_1* | 1.10E-09 | 4.80E-08 | NS | NS | 1.80E-07 | 4.80E-08 | NS |
| *Firmicutes_Lachnospiraceae_Roseburia-a* | 7.44E-11 | 7.84E-07 | NS | 0.01069 | 7.84E-07 | 1.47E-07 | 0.0001755 |
| *Firmicutes_Lachnospiraceae_Roseburia-b* | 2.17E-09 | NS | NS | 4.80E-08 | NS | 6.76E-08 | 4.80E-08 |
| *Firmicutes_Lachnospiraceae_Ruminococcus_torques_group-a* | 2.55E-10 | 1.47E-05 | 0.000401 | 0.000401 | 4.80E-08 | 4.80E-08 | NS |
| *Firmicutes_Lachnospiraceae_Ruminococcus_torques_group-b* | 1.13E-11 | 4.80E-08 | NS | 4.80E-08 | 1.80E-07 | NS | 1.80E-07 |
| *Firmicutes_Lactobacillaceae_Lactobacillus-a* | 2.21E-09 | NS | NS | 1.18E-07 | NS | 6.76E-08 | 2.77E-07 |
| *Firmicutes_Lactobacillaceae_Lactobacillus-b* | 2.54E-07 | 3.04E-06 | 7.20E-06 | 2.33E-06 | NS | NS | NS |
| *Firmicutes_Ruminococcaceae_Faecalibacterium* | NS | NA | NA | NA | NA | NA | NA |
| *Firmicutes_Ruminococcaceae_Ruminococcus_2* | NS | NA | NA | NA | NA | NA | NA |
| *Firmicutes_Staphylococcaceae_Staphylococcus* | 2.92E-02 | NS | NS | NS | 0.04177 | NS | NS |
| *Firmicutes_Streptococcaceae_Streptococcus_pneumoniae* | NS | NA | NA | NA | NA | NA | NA |
| *Firmicutes_Streptococcaceae_Streptococcus-a* | NS | NA | NA | NA | NA | NA | NA |
| *Firmicutes_Streptococcaceae_Streptococcus-b* | NS | NA | NA | NA | NA | NA | NA |
| *Firmicutes_Veillonellaceae_Veillonella* | NS | NA | NA | NA | NA | NA | NA |
| *Proteobacteria_Alcaligenaceae_Parasutterella-a* | 1.57E-11 | 2.78E-07 | 0.008476 | 1.80E-07 | 2.78E-07 | NS | 1.80E-07 |
| *Proteobacteria_Alcaligenaceae_Parasutterella-b* | 2.17E-09 | NS | 6.76E-08 | NS | 6.76E-08 | NS | 4.80E-08 |
| *Proteobacteria_Alcaligenaceae_Sutterella* | 8.75E-13 | 1.47E-07 | 1.15E-06 | 4.80E-08 | 7.84E-07 | NS | 1.80E-07 |
| *Proteobacteria_Desulfovibrionaceae_Desulfovibrio* | 3.28E-10 | 0.003377 | NS | 2.09E-07 | 0.02591 | 2.09E-07 | 7.39E-06 |
| *Proteobacteria_Enterobacteriaceae_Escherichia/Shigella* | 2.85E-10 | 3.62E-07 | 0.004318 | NS | 9.09E-08 | 1.77E-07 | NS |
| *Proteobacteria_Enterobacteriaceae_Klebsiella* | 4.56E-04 | 0.000154 | NS | NS | 0.000154 | 0.000154 | 0 |
| *Proteobacteria_Neisseriaceae_Neisseria* | NS | NA | NA | NA | NA | NA | NA |
| *Proteobacteria_Pasteurellaceae_Haemophilus* | NS | NA | NA | NA | NA | NA | NA |
| *Verrucomicrobia_Verrucomicrobiaceae_Akkermansia* | 8.39E-12 | NS | 1.47E-07 | 1.47E-07 | 1.74E-06 | 4.18E-06 | NS |

**Supplemental Table 2.** Measurements at peak responses of resistance (Rrs in cmH2O.s/ml), compliance (Crs in ml/cmH2O), elastance (Ers in cmH2O/ml), tissue elastance (H in, cmH2O/ml), tissue damping (G in cmH2O/ml), and conducting airway (Newtonian) resistance (Rn in cmH2O.s/ml) for each methacholine dose for phosphate buffered saline (PBS) treatment groups. The peak response is the top three measurements for all parameters except Crs, for which it is the lowest three measurements. *x ± s* represents average ± 1 SD. N is the number of non-missing values.

|  | **Methacholine dose** | | | | |
| --- | --- | --- | --- | --- | --- |
| **Lung function parameter** | **0 mg/ml**  (N=36) | **12.5 mg/ml**  (N=36) | **25 mg/ml**  (N=36) | **50 mg/ml**  (N=36) | **100 mg/ml**  (N=36) |
| **Baseline Rrs** | **0.533 ± 0.097** | **^†^0.562 ± 0.096^*^** | **^†^0.853 ± 0.196** | **^†^0.980 ± 0.243** | **^†^1.215 ± 0.31** |
| **Peak Respiratory Resistance (Rrs)** | **0.59 ± 0.10** | **0.96 ± 0.22** | **1.54 ± 0.47** | **1.67 ± 0.58** | **2.27 ± 1.29** |
| **Baseline_Crs** | **0.0374 ± 0.0085** | **^†^0.0355 ± 0.007** | **^†^0.0316 ± 0.008** | **^†^0.0290 ± 0.007** | **^†^0.0267 ± 0.007** |
| **Peak Respiratory Compliance (Crs)** | **0.0353 ± 0.0077** | **0.0305 ± 0.0077** | **0.0260 ± 0.0074** | **0.0252 ± 0.0081** | **0.0200 ± 0.0082** |
| **Baseline_Ers** | **27.9 ± 5.6** | **^†^29.4 ± 5.8** | **^†^33.4 ± 7.2** | **^†^36.3 ± 8.0** | **^†^40.2 ± 10.4** |
| **Peak Respiratory Elastance**  **(Ers)** | **29.5 ± 5.8** | **^†^34.5 ± 7.4** | **^†^41.2 ± 10.3** | **^†^43.2 ± 12.6** | **^†^58.4 ± 24.2** |
| **Baseline_H** | **25.4 ± 5.3** | **^†^26.4 ± 5.7** | **^†^27.9 ± 6.3** | **^†^30.2 ± 7.1** | **^†^32.6 ± 10.6** |
| **Peak Tissue Elastance**  **(H)** | **27.3 ± 5.6** | **29.5 ± 6.4** | **31.5 ± 7.2** | **34.6 ± 10.6** | **42.1 ± 13.9** |
| **Baseline_G** | **4.29 ± 0.85** | **^†^4.33 ± 0.77** | **^†^5.48 ± 1.27** | **^†^5.79 ± 1.38** | **^†^7.29 ± 3.19** |
| **Peak Tissue Resistance**  **(G)** | **4.77 ± 0.78** | **6.27 ± 1.42** | **8.97 ± 2.59** | **9.48 ± 3.57** | **12.11 ± 5.50** |
| **Baseline_Rn** | **0.202 ± 0.048** | **^†^0.217 ± 0.056** | **^†^0.358 ± 0.095** | **^†^0.460 ± 0.146** | **^†^0.599 ± 0.189** |
| **Peak Conducting Airway Resistance**  **(Rn)** | **0.256 ± 0.051** | **0.437 ± 0.093** | **0.679 ± 0.184** | **0.855 ± 0.253** | **1.071 ± 0.402** |

^†^ Baselines for doses 12.5-100 mg/ml of Mch correspond to the last value measured by *flexivent* at the end of the dose response curve of the preceding dose.

**Supplemental Table 3.** Measurements at peak responses of resistance (Rrs in cmH2O.s/ml), compliance (Crs in ml/cmH2O), elastance (Ers in cmH2O/ml), tissue elastance (H in, cmH2O/ml), tissue damping (G in cmH2O/ml), and conducting airway (Newtonian) resistance (Rn in cmH2O.s/ml) for each methacholine dose for house dust mite (HDM) treatment groups. The peak response is the top three measurements for all parameters except Crs, for which it is the lowest three measurements. *x ± s* represents average ± 1 SD. N is the number of non-missing values.

|  | **Methacholine dose** | | | | |
| --- | --- | --- | --- | --- | --- |
| **Lung function parameter** | **0 mg/ml**  **(N=40)** | **12.5 mg/ml**  **(N=40)** | **25 mg/ml**  **(N=40)** | **50 mg/ml**  **(N=40)** | **100 mg/ml**  **(N=40)** |
| **Baseline_Rrs** | **0.56 ± 0.11** | **^†^0.60 ± 0.13** | **^†^0.95 ± 0.35** | **^†^1.37 ± 0.67** | **^†^1.82 ± 0.85** |
| **Peak Respiratory Resistance**  **(Rrs)** | **0.64 ± 0.21** | **1.04 ± 0.38** | **1.93 ± 1.25** | **2.55 ± 1.14** | **3.97 ± 3.81** |
| **Baseline_Crs** | **0.0365 ± 0.0081** | **^†^0.0342 ± 0.0080** | **^†^0.0297 ± 0.0075** | **^†^0.0255 ± 0.0083** | **^†^0.0224 ± 0.0078** |
| **Peak Respiratory Compliance**  **(Crs)** | **0.0339 ± 0.0076** | **0.0289 ± 0.0077** | **0.0232 ± 0.0083** | **0.0202 ± 0.0078** | **0.0152 ± 0.0073** |
| **Baseline_Ers** | **28.8 ± 7.0** | **^†^31.1 ± 8.4** | **^†^36.3 ± 11.2** | **^†^45.0 ± 20.0** | **^†^51.9 ± 23.5** |
| **Total Respiratory Elastance**  **(Ers)** | **31.3 ± 8.3** | **37.9 ± 13.7** | **52.2 ± 31.6** | **58.8 ± 27.0** | **90.3 ± 61.8** |
| **Baseline_H** | **26.6 ± 7.0** | **^†^28.4 ± 8.7** | **^†^30.5 ± 10.0** | **^†^34.4 ± 13.0** | **^†^37.7 ± 16.3** |
| **Peak Tissue Elastance**  **(H)** | **29.9 ± 8.8** | **32.0 ± 9.9** | **37.0 ± 15.2** | **42.1 ± 18.6** | **52.2 ± 23.2** |
| **Baseline_G** | **4.6 ± 1.1** | **^†^4.8 ± 1.1** | **^†^6.1 ± 2.2** | **^†^7.8 ± 3.8** | **^†^9.9 ± 4.7** |
| **Peak Tissue Damping**  **(G)** | **5.3 ± 1.5** | **6.8 ± 2.5** | **11.4 ± 7.0** | **13.6 ± 6.5** | **20.0 ± 14.4** |
| **Baseline_Rn** | **0.204 ± 0.062** | **^†^0.223 ± 0.061** | **^†^0.387 ± 0.150** | **^†^0.569 ± 0.197** | **^†^0.769 ± 0.338** |
| **Peak Conducting Airway Resistance**  **(Rn)** | **0.260 ± 0.056** | **0.474 ± 0.180** | **0.746 ± 0.291** | **1.115 ± 0.429** | **1.332 ± 0.719** |

^†^ Baselines for doses 12.5-100 mg/ml of Mch correspond to the last value measured by *flexivent* at the end of the dose response curve of the preceding dose.

**Supplemental Table 4.** Linear mixed effects model for respiratory system resistance (Rrs) data: type III sum of squares and contrasts corresponding to significant categorical variables. *P-values adjusted with Tukey method; significant values are bolded.

| **Variable** | $\boldsymbol{\chi}^{\boldsymbol{2}}-$**statistic** | **Degrees of freedom** | **P-value** |
| --- | --- | --- | --- |
| Intercept | 30.4520 | 1 | 0.0000 |
| Log(Baseline_Rrs) | 353.3577 | 1 | 0.0000 |
| Dose | 0.4688 | 1 | 0.4935 |
| Microbiota | 1.7320 | 3 | 0.6298 |
| Treatment | 1.2579 | 1 | 0.2621 |
| Dose*Microbiota | **9.1195** | **3** | **0.0277*** |
|  | **Estimates of dose effects for microbiota groups** | | |
|  | **Estimate** | **Standard Error** | **95% Conf. Interval (Slope)** |
| Mouse | 0.0009 | 0.0013 | (-0.0016,0.0033) |
| Human Adult | 0.0039 | 0.0014 | (0.0012,0.0067) |
| Infant A | 0.0049 | 0.0016 | (0.0019,0.0080) |
| Infant B | 0.0067 | 0.0019 | (0.0031,0.0104) |
|  | **Contrasts of dose effects between different microbiota groups** | | |
|  | **Estimate** | **Standard Error** | **P-value** |
| Human Adult - Mouse | 0.0031 | 0.0016 | 0.2365 |
| Infant A - Mouse | 0.0041 | 0.0017 | 0.0778 |
| Infant B - Mouse | **0.0059** | **0.0019** | **0.0119*** |
| Infant A - Human Adult | 0.0010 | 0.0018 | 0.9433 |
| Infant B - Human Adult | 0.0028 | 0.0020 | 0.4813 |
| Infant B - Infant A | 0.0018 | 0.0020 | 0.7900 |

**Supplemental Table 5.** Pearson correlation coefficients (ρ) for baselines of each of the lung function parameters versus total serum IgE and separated by treatment. “Corr” value corresponds to the correlation value for all microbiota groups given that specific treatment. (PBS or HDM). Correlation coefficients values separated by microbiota are shown below. A value between 0.3 to 0.5 is considered moderate positive correlation, while a value between 0.5 to 1.0 is considered strong positive correlation. *- indicates correlation test p values that were <0.05. ** - indicates correlation test p values that were <0.1.

|  | **Total Serum IgE** | |
| --- | --- | --- |
|  | **PBS (ρ)** | **HDM (ρ)** |
| **Total airway resistance (Rrs)** | **Corr: 0.408***  Mouse: 0.655*  Adult C: 0.409  INF A: 0.644  INF B: 0.394 | **Corr: 0.650*****  Mouse: −0.066  Adult C: 0.772**  INF A: 0.544  INF B: 0.201 |
| **Total airway Compliance (Crs)** | **Corr: −0.326**  Mouse: −0.378  Adult C: −0.161  INF A: −0.565  INF B: −0.387 | **Corr: −0.390***  Mouse: −0.266  Adult C: −0.467  INF A: −0.383  INF B: 0.125 |
| **Total airway elastance (Ers)** | **Corr: 0.342***  Mouse: 0.393  Adult C: 0.160  INF A: 0.680*  INF B: 0.273 | **Corr: 0.460****  Mouse: 0.266  Adult C: 0.537  INF A: 0.349  INF B: −0.214 |
| **Central airways resistance (Rn)** | **Corr: 0.103**  Mouse: 0.347  Adult C: −0.180  INF A: −0.354  INF B: 0.300 | **Corr: 0.339***  Mouse: −0.112  Adult C: 0.604*  INF A: 0.282  INF B: 0.058 |
| **Tissue resistance (G)** | **Corr: 0.367***  Mouse: 0.246  Adult C: 0.513  INF A: 0.699.  INF B: −0.078 | **Corr: 0.423****  Mouse: 0.063  Adult C: 0.510  INF A: 0.416  INF B: −0.308 |
| **Tissue Elastance (H)** | **Corr: 0.245**  Mouse: 0.388  Adult C: −0.185  INF A: 0.793*  INF B: 0.428 | **Corr: 0.423****  Mouse: 0.063  Adult C: 0.510  INF A: 0.416  INF B: −0.308 |
